# Delivery of Rapamycin Using *In Situ* Forming Implants Promotes Immunoregulation and Vascularized Composite Allograft Survival

**DOI:** 10.1038/s41598-019-45759-y

**Published:** 2019-06-25

**Authors:** Damian Sutter, Dzhuliya V. Dzhonova, Jean-Christophe Prost, Cedric Bovet, Yara Banz, Lisa Rahnfeld, Jean-Christophe Leroux, Robert Rieben, Esther Vögelin, Jan A. Plock, Paola Luciani, Adriano Taddeo, Jonas T. Schnider

**Affiliations:** 1 Department of Plastic and Hand Surgery, Inselspital, Bern University Hospital, University of Bern, Bern, Switzerland; 2 Department for BioMedical Research, University of Bern, Bern, Switzerland; 3 University Institute of Clinical Chemistry, Inselspital, Bern University Hospital, University of Bern, Bern, Switzerland; 4 Institute of Pathology, University of Bern, Bern, Switzerland; 5 Department of Pharmaceutical Technology, Institute of Pharmacy, University of Jena, Jena, Germany; 6 Institute of Pharmaceutical Sciences, Department of Chemistry and Applied Biosciences, ETH Zürich, Zürich, Switzerland; 7 Department of Plastic Surgery and Hand Surgery, University Hospital Zurich, University of Zurich, Zürich, Switzerland; 8 Present Address: Department of Chemistry and Biochemistry, University of Bern, Bern, Switzerland

**Keywords:** Allotransplantation, Translational research

## Abstract

Vascularized composite allotransplantation (VCA), such as hand and face transplantation, is emerging as a potential solution in patients that suffered severe injuries. However, adverse effects of chronic high-dose immunosuppression regimens strongly limit the access to these procedures. In this study, we developed an *in situ* forming implant (ISFI) loaded with rapamycin to promote VCA acceptance. We hypothesized that the sustained delivery of low-dose rapamycin in proximity to the graft may promote graft survival and induce an immunoregulatory microenvironment, boosting the expansion of T regulatory cells (T_reg_). *In vitro* and *in vivo* analysis of rapamycin-loaded ISFI (Rapa-ISFI) showed sustained drug release with subtherapeutic systemic levels and persistent tissue levels. A single injection of Rapa-ISFI in the groin on the same side as a transplanted limb significantly prolonged VCA survival. Moreover, treatment with Rapa-ISFI increased the levels of multilineage mixed chimerism and the frequency of T_reg_ both in the circulation and VCA-skin. Our study shows that Rapa-ISFI therapy represents a promising approach for minimizing immunosuppression, decreasing toxicity and increasing patient compliance. Importantly, the use of such a delivery system may favor the reprogramming of allogeneic responses towards a regulatory function in VCA and, potentially, in other transplants and inflammatory conditions.

## Introduction

Vascularized composite allotransplantation (VCA) has become a clinical reality during the last decade, and has been increasingly evaluated as a therapeutic reconstructive option for patients who have suffered extensive facial injuries or debilitating hand amputation^1^. In particular, hand transplants have been successfully performed with excellent functional and esthetic outcomes by several centers around the world^2^. However, long-term adverse effects of immunosuppressive treatment prevent a wider clinical application of this “life-enhancing” rather than “life-saving” procedure. Unlike solid organ transplantation, VCA offers unique opportunities for local delivery of immunosuppressive agents directly to the graft^3^. We and other groups have shown that site-specific immunosuppression can be successfully used in VCA employing topical FK506^4,5^ and clobetasol^5^, hydrogel-based drug delivery systems laden with FK506^6,7^, intra-graft injections of FK506^8^, and biodegradable disks containing FK506-loaded microspheres^9^. All these approaches aim to reduce systemic exposure and global collateral or end-organ adverse effects while maintaining therapeutic levels in the different tissues of the grafts, especially skin.

Importantly, drugs administered directly into the graft may not only reduce potential side effects but also directly influence the magnitude and nature of an allogeneic immune response by promoting immune-regulation through the expansion of donor-specific regulatory T cells (T_reg_)^10^. Indeed, accumulating evidence suggests that graft rejection is ultimately determined by the balance between allo-aggressive T cells and allospecific T_reg_ enabling donor-specific tolerance^11^. Hence, several groups have focused their efforts on optimizing therapeutic protocols aimed at inducing allospecific T_reg_ which are able to mitigate the immunoresponse to engraftment^12^.

Rapamycin is a macrolide antibiotic structurally similar to FK506. It binds to FK506 Binding Protein-12 and affects the G1 phase of the cell cycle by acting on a unique cellular target called mammalian target of rapamycin (mTOR)^13^. Recently, it has been demonstrated that, in contrast to cyclosporine and FK506, rapamycin can promote differentiation of T_reg_ both *in vivo* and *in vitro* while blunting Th17 differentiation and function^14–17^. Moreover, a significant increase in T_reg_ numbers has been reported in kidney transplant patients under rapamycin therapy when compared to treatment with calcineurin inhibitors^17–21^.

In this study, we developed an innovative drug delivery system that combines the advantage of *in situ* delivery with the potential to induce local immune-regulation and thus transplant survival. To this aim, we designed a solvent-induced phase inversion *in situ* forming implant (ISFI) using the US Food and Drug Administration approved polymer poly(D,L-lactic-co-glycolic acid) (PLGA). We loaded this ISFI with the immunoregulatory drug rapamycin and injected it in close proximity to the transplant. We hypothesized that sustained low-dose delivered rapamycin may promote graft survival with minimal immunosuppression through the induction of immunoregulatory mechanisms such as T_reg_ expansion and increased chimerism levels.

## Results

### Design and drug release properties of ISFI

**Figure 1:**
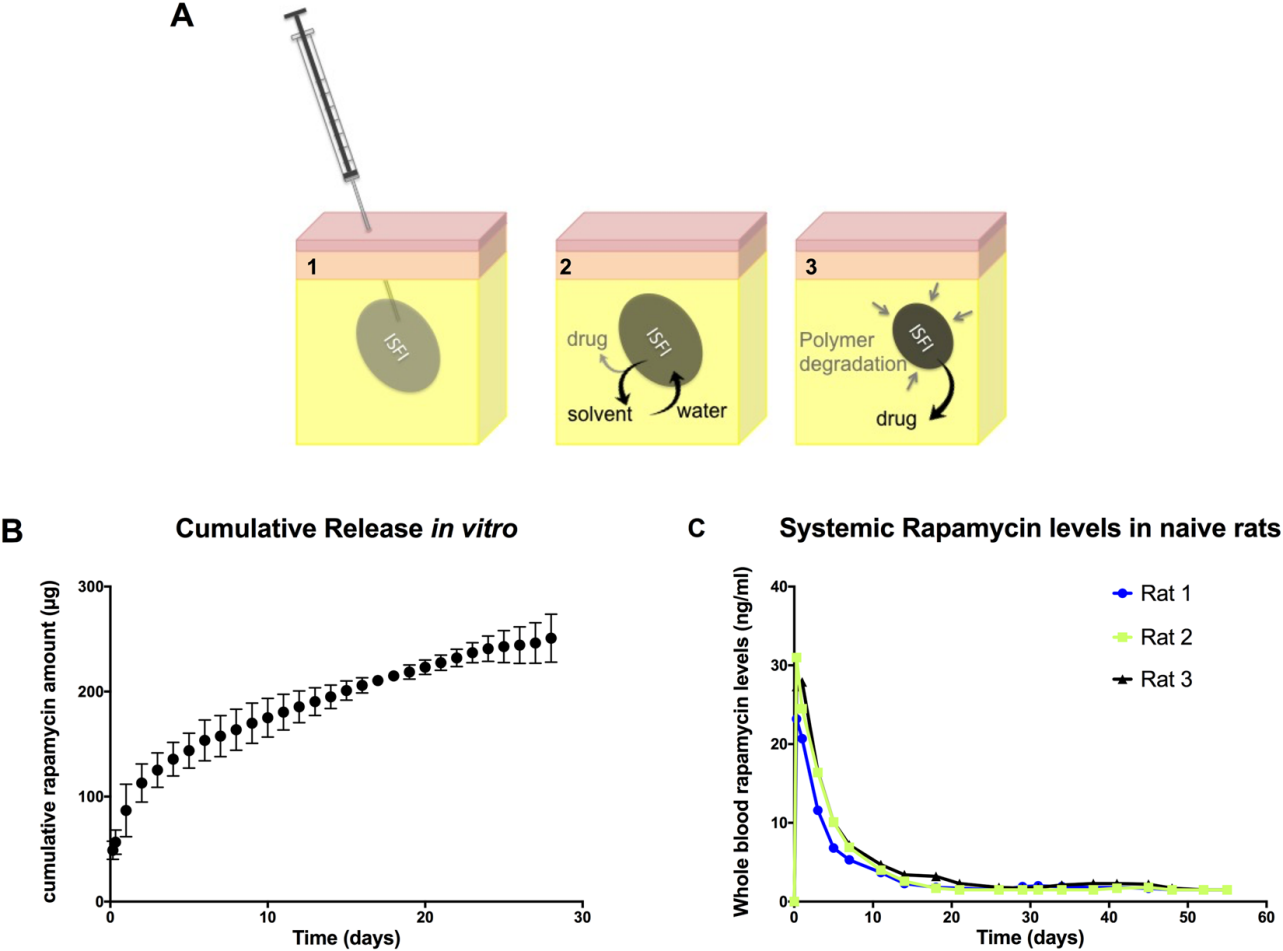
Design and evaluation of rapamycin-loaded ISFI (Rapa-ISFI). (**A**) Schematic representation of the Rapa-ISFI formation and drug release properties. (1) Upon injection into the subcutaneous tissue, (2) the biocompatible solvent *N*-methyl-2-pyrrolidone (NMP) diffuses out of solution into the surrounding tissue, causing the biocompatible and biodegradable PLGA-polymer to solidify in the aqueous environment of the interstitial tissue, trapping the drug within. Since the drug is soluble in NMP, a certain amount of drug will evade entrapment in the solid implant and account for an initial burst release. (3) As the implant is degraded over time, the drug is then released gradually. The drug can also be released via diffusion through the polymer matrix. (**B**) *In vitro* analysis of rapamycin release from Rapa-ISFI. Rapa-ISFI were prepared and injected into stainless steel mesh baskets suspended in release medium and rapamycin was quantified using high-performance liquid chromatography at different time point. The cumulative amount of rapamycin (total µg in the solution) is reported for the different sampling times. The displayed data are mean ± standard deviation (S.D.) of three independent samples. (**C**) *In vivo* rapamycin release from Rapa-ISFI. Three naïve Lewis rats were injected subcutaneously in one hind limb groin with Rapa-ISFI. Blood was sampled at designated time points and rapamycin concentration was measured by LC-MS/MS.

Rapa-ISFI loaded with 5 mg rapamycin were formulated and tested *in vitro* and *in vivo*. A schematic representation of the ISFI is presented in Fig. 1A. The *in vitro* release kinetics showed a small initial burst during the first 24 h, which could be attributed to the release of the drug during implant formation and to the surface associated drug. The release was sustained for the first 6 days and 3.07 ± 0.39% of the drug was released during this initial phase of burst-release. This is typical of PLGA implant and attributed to the bulk degradation of the system^22,23^. From day 7 until the end of the experiment (ca. 1 month) the release rate was sustained at 4.43 ± 1.24 µg/d (Fig. 1B). *In vivo* studies in naïve rats showed a release pattern comparable to the *in vitro* results. A burst release was observed within the first 24 h, reaching a blood concentration of 27 ± 4 ng/mL. Systemic levels decreased gradually reaching levels below 5 ng/mL within 11 days. Thereafter, subtherapeutic systemic levels (range 1.8–1.5 ng/mL) were measurable up to 48 days (Fig. 1C). Notably, Rapa-ISFI solidified promptly after subcutaneous injection forming a solid depot without spreading outside the injection site (Supplementary Fig. S1). No inflammation was observed at the injection site and the depot was evident and palpable for about three weeks. After this period, Rapa-ISFI could be detected for about another two weeks only by palpation and then it became undetectable and the implant could not be found in any rat sacrificed later than 40 days after Rapa-ISFI injection.

### Rapamycin-loaded ISFI promote VCA survival

**Figure 2:**
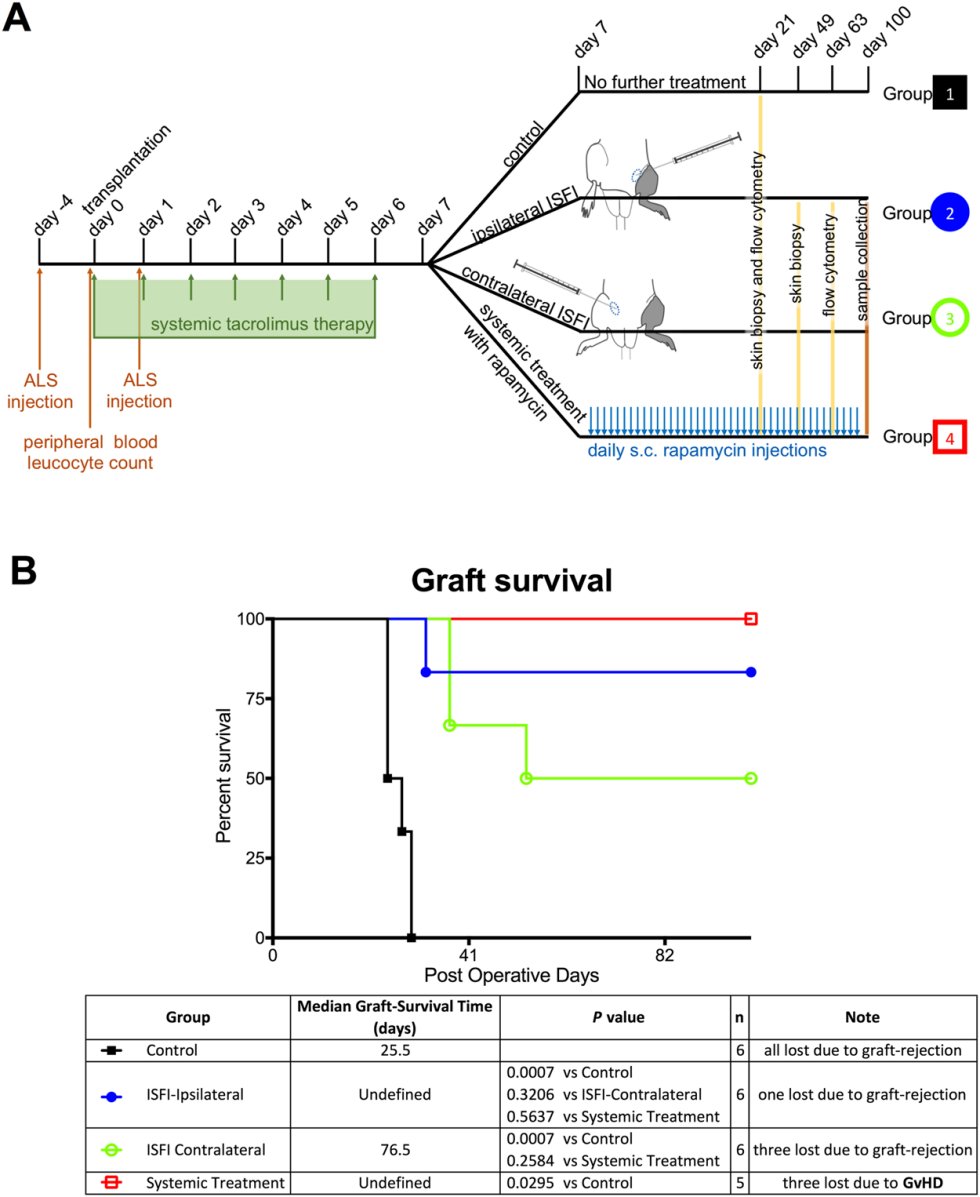
Rapa-ISFI treatment prolonged survival of vascularized composite allografts. (**A**) Experimental design. (**B**) Graft survival represented with Kaplan-Meier survival curves. (Group 1, n = 6; Group 2, n = 6; Group 3, n = 6; Group 4, n = 5). Median Survival time (MST) refers specifically to graft survival. The appearance of GvHD in three out of five rats of group 4 and the P value calculated by Mantel-Cox test are reported for each group.

To assess the effects of Rapa-ISFI treatment on the survival of a fully MHC-mismatched VCA, we performed Brown Norway-to-Lewis hind limb transplantation. The experimental protocol is shown in Fig. 2A. Untreated hind limb allografts (Group 1, control) were rejected with 25.5 days median survival time (MST). In Group 2 (Rapa-ISFI injected on the ipsilateral side, see Supplementary Fig. S1), 83.3% of the rats reached POD100 with an allograft MST >100 days (p = 0.0007 versus Group 1) (Fig. 2B). Within Group 2, one rat rejected at POD32; one rat progressed to grade 2 rejection at POD28 and remained at this stage until the endpoint; two rats showed grade 1 rejection at POD21 followed by spontaneous resolution of the rejection episode; one rat showed no signs of rejection during the experiment (see Supplementary Fig. S2). Injection of Rapa-ISFI into the contralateral limb significantly prolonged graft survival with 50% of the rats reaching POD100 and a MST of 76.5 days (p = 0.007 versus Group 1 and p = 0.33 versus Group 2) (Fig. 2B). In this group, three rats rejected their limbs; one rat showed a grade 2 rejection episode at POD30 that reverted to grade 0 at POD73 and the other two rats showed no signs of rejection during the entire experiment (see Supplementary Fig. S2). In the group treated with daily injection of rapamycin (Group 4, systemic treatment), 3 out of 5 rats (60%) developed clear signs of acute GvHD and had to be sacrificed between POD33 and 41. The other two rats (40%) reached the endpoint without signs of GvHD or graft rejection. Median graft survival time was 100 days, significantly higher than control animals (p = 0.0295 versus Group 1) and without significant differences as compared to Rapa-ISFI-treated animals (Fig. 2B). However, general animal survival of Group 4 was 41 days, due to GvHD development. Specifically, macroscopic signs of GvHD started to appear around POD21 and included ear dermatoerythema, diarrhea and tongue lesions. To further confirm the development of GvHD, we analyzed injured ears and tongues by flow cytometry, revealing infiltration of donor T cells in GvHD lesions (see Supplementary Fig. S3). Moreover, in these animals, we observed donor-specific hyporesponsiveness but normal response to third party stimulation at POD21 in an *in vitro* MLR assay (see Supplementary Fig. S4).

**Figure 3:**
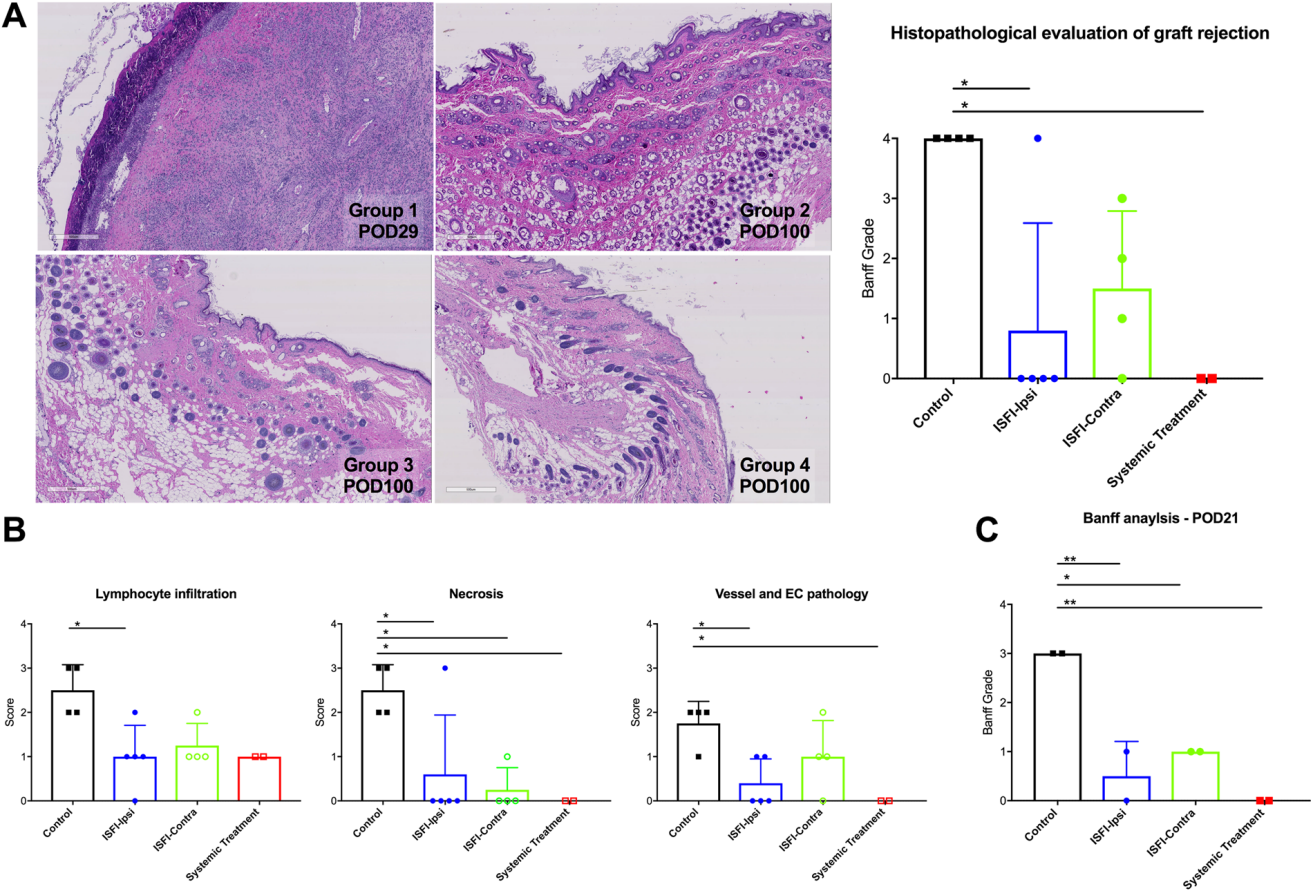
Histological evaluation of the different treatments. (**A**) Representative microphotographs of the histology sections of the skin stained with hematoxylin and eosin and histopathological grading of rejection based on Banff working classification for VCA rejection^24^ in the 4 treatment groups. Skin was recovered from all the allografts at rejection or at the endpoint. Rats of Group 4 that developed lethal GvHD were excluded from the analysis. (**B**) Specific assessment of leukocyte infiltration, tissue necrosis and vascular, including endothelial cell (EC) pathology in the 4 treatment groups. For each of these categories a score from 0 to 3 was given (*i*.*e*., 0 = absent, 1 = minimal, 2 = moderate or 3 = extensive). (**C**) Histopathological grading of rejection based on Banff working classification for VCA rejection^24^ in the 4 treatment groups at post-operative day (POD) 21 (n = 2 per group). Data are presented as mean and SD. *P < 0.05, **P < 0.01 by one-way ANOVA with Tukey’s multi-comparisons test.

Histological grading of rejection based on the Banff working classification^24^ confirmed the macroscopic findings. As compared to rats of Group 1, we observed a significant reduction of Banff score in rats from Group 2, with reduction of lymphocyte infiltration, tissue necrosis and vascular pathology (Fig. 3A,B). Conversely, allografts from Group 3 only showed a significant reduction of tissue necrosis. Finally, in the two rats of Group 4 without GvHD we observed minimal tissue damage with only moderate lymphocytic infiltration. Interestingly, histological evaluation of skin biopsies retrieved at POD21 (i.e. before rejection occurred in the control group) anticipated the changes observed at the end point, with a significantly lower Banff score in the rats from all the groups as compare to rats from Group 1 (Banff score 0.5 ± 0.7, 1 ± 0 and 0 ± 0 in Group 2, 3 and 4, respectively, versus 3 ± 0 in Group 1, p = 0.0073, p = 0.0164 and p = 0.0037, respectively) (Fig. 3C). Muscle histopathology revealed only mild tissue damage upon rejection with minimal leukocyte infiltration and muscle necrosis and/or muscle atrophy. Rapamycin-treated allografts presented a tendency to reduced muscle pathology as compared to untreated rats (see Supplementary Fig. S5).

### Rapamycin levels in hind limb transplanted rats

**Figure 4:**
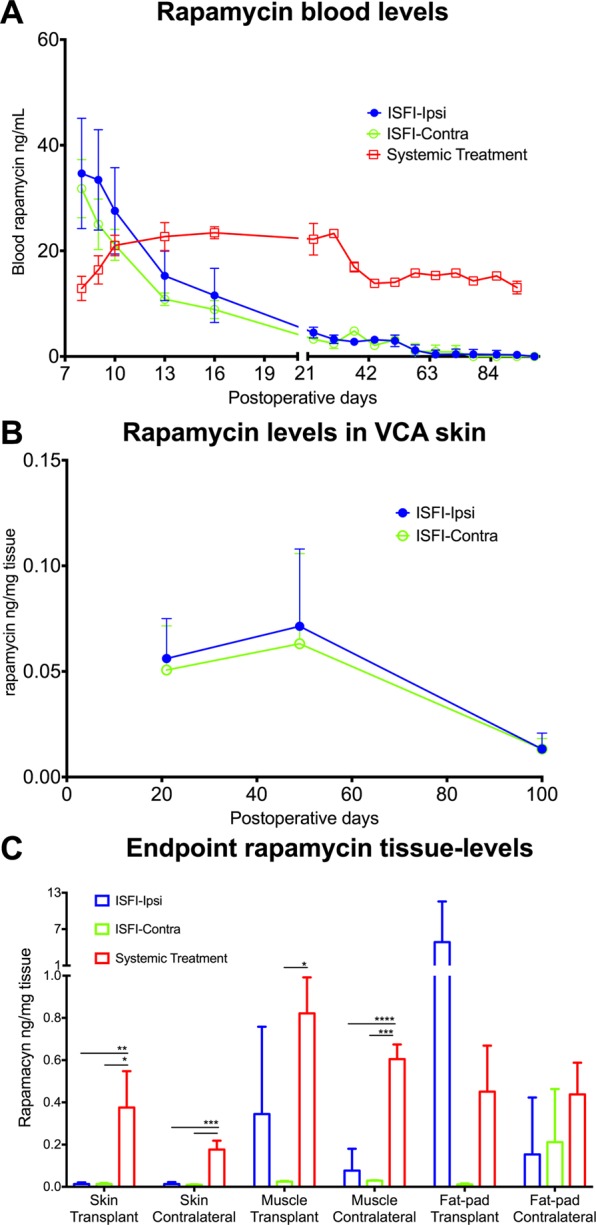
Systemic and tissue levels of rapamycin. (**A**) Whole blood levels of rapamycin in rats from Group 2 (n = 4–6), Group 3 (n = 1–5) and Group 4 (n = 1–5). Rapamycin levels were measured by LC-MS/MS in the blood at different postoperative days (POD) and expressed as ng rapamycin per mL of blood. For Group 4 trough concentrations are shown (*i*.*e*., blood collected about 18–24 h after systemic rapamycin injection). (**B**) Skin rapamycin levels in rats treated with Rapa-ISFI. Skin biopsies were recovered from the allograft 21 (n = 2 for Group 2 and n = 4 for Group 3), 49 (n = 2 for group 2 and n = 2 for group 3) and 100 days (n = 5 for Group 2 and n = 2 for Group 3) after transplantation. Data presented as ng of rapamycin per mg of tissue. (**C**) Rapamycin levels in different tissues at the endpoint of long-term surviving rats of different treatment groups. Rapamycin was measured in skin, muscle and groin fat-pad recovered from the transplant side or the contralateral control limb. Data presented as mean and SD, *P < 0.05, **P < 0.01, ***P < 0.001, ****P < 0.0001 by one-way ANOVA with Tukey’s multi-comparisons test. Rats from Group 1 were also tested as a negative control and they show rapamycin levels under the quantification limit both in blood and tissue (not shown).

As shown in Fig. 4A, systemic daily injections of rapamycin (Group 4) generated an average trough concentration of 17.3 ± 3.9 ng/mL of drug (range 12.9–23.3 ng/mL). In transplanted animals that received a Rapa-ISFI, we observed an initial burst release of rapamycin with systemic blood levels at POD8 of 34.7 ± 10.4 and 31.8 ± 5.5 ng/mL in Groups 2 and 3, respectively (p = 0.6351, by unpaired *t* test). After this, the levels decreased to 4.5 ± 1.0 and 3.3 ± 0.8 ng/mL at POD23 (Group 2 and 3, respectively, p = 0.0746) and remained constant until POD58. Afterwards, the levels dropped below the quantification limit (*i*.*e*. 1.5 ng/mL).

In order to measure the tissue levels of rapamycin in the transplant, skin biopsies were analyzed at POD21, 49 and 100 in Groups 2 and 3. The injection of an ISFI either in the ipsilateral or in the contralateral limb generated VCA-skin concentrations of 0.06 ± 0.2 and 0.05 ± 0.02 ng/mg of tissue, respectively at POD21 (p = 0.7729). The levels reached 0.07 ± 0.04 and 0.06 ± 0.04 ng/mg of tissue at POD 49 (p = 0.8547), and then at endpoint dropped to 0.01 ± 0.01 and 0.01 ± 0.01 ng/mg, respectively (Fig. 4B).

At the endpoint, skin, muscle and fat-pad tissues from the transplanted side and the contralateral side were recovered and analyzed for rapamycin concentrations. Similar levels of rapamycin were observed in skin and muscle (Fig. 4C) of rats injected with Rapa-ISFI, independent of the Rapa-ISFI injection site (i.e. Groups 2 and 3) and tissue collection site (i.e. transplanted or contralateral limb). Fat pad levels showed high variation in the transplanted side of Group 2 but they were not significantly different as compared to Group 3. In the contralateral fat pad, values were similar in Groups 2 and 3. Systemically treated rats (Group 4) presented tissue levels of rapamycin in skin and muscle significantly higher as compared to Groups 2 and 3 (Fig. 4C). However, in this group we observed uniform rapamycin tissue concentrations (average among the tissues was 0.48 ± 0.22 ng/mg) with no significant difference between tissues retrieved from the transplanted or contralateral side, suggesting that the differences observed in Groups 2 and 3 are connected to the persistent drug-release rather than accumulation or different metabolism rate.

### Rapamycin treatment promotes multilineage mixed chimerism

**Figure 5:**
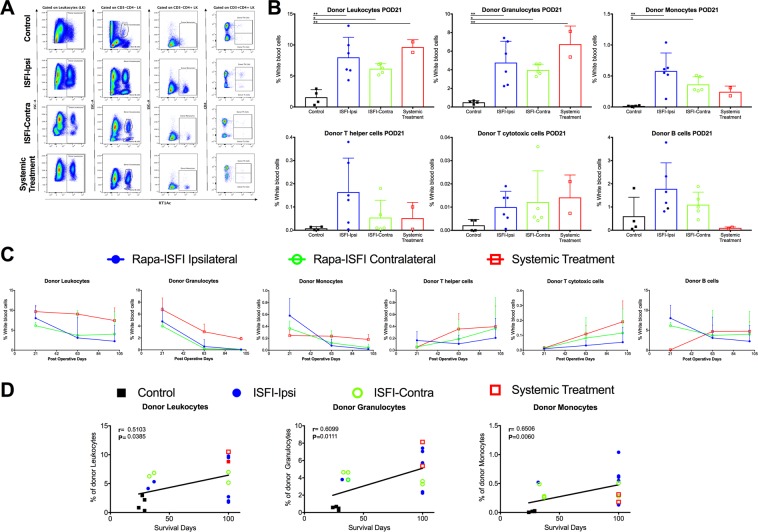
Rapamycin treatment promotes multilineage mixed chimerism. (**A**,**B**) Multilineage mixed chimerism levels at POD21 in the peripheral blood of the rats of different treatment groups. (**A**) Representative flow cytometry for the quantification of multilineage chimerism in the four groups (representative gating strategy presented in Supplementary Fig. 6). Donor leukocytes were identified as RT1Ac^+^ cells in the leukocytes gate; donor granulocytes as CD3^−^CD4^−^SSc^High^RT1Ac^+^ leukocytes; donor monocytes as CD3^−^CD4^+^RT1Ac^+^ leukocytes; donor T helper (Th) cells as CD3^+^CD4^+^RT1Ac^+^ leukocytes; donor T cytotoxic (Tc) cells as CD3^+^CD4^−^RT1Ac^+^ leukocytes and donor B cells as CD3^−^CD4^−^ SSc^Low^RT1Ac^+^ leukocytes. (**B**) Quantitative summaries of multilineage chimerism quantification at POD21 in the four groups. Data presented as mean and SD, *P < 0.05, **P < 0.01 by one-way ANOVA with Tukey’s multi-comparisons test. (**C**) Evolution of multilineage mixed chimerism. Flow cytometry analysis for measuring the frequency of donor cells was performed at POD21 (same data of Fig. 4A), 63 and 100 in the rats from Groups 2 (n = 5), 3 (n = 5 POD21 and n = 3 POD63 and 100) and 4 (n = 2). (**D**) Correlation analysis between chimerism levels and allograft survival. Frequency of donor leukocytes, granulocytes and monocytes in peripheral blood of rats from Group 1 (closed squares), Group 2 (closed circles), Group 3 (open circles) and Group 4 (open squares) at POD21 were correlated with allograft survival by nonparametric (Spearman) correlation, r values and P values are reported for each correlation.

To verify whether Rapa-ISFI treatment influenced the levels of mixed chimerism, we measured the frequency of donor cells in the peripheral blood of recipient rats at different time points by flow cytometry (see Supplementary Fig. S6 for a representative gating strategy). At first, we focused our analysis on POD21, which allowed for comparison of all 4 groups two weeks after the end of the bridging therapy and the start of specific treatments. As shown in Fig. 5A, treatment with rapamycin in Groups 2, 3 and 4 was associated with higher frequency of RT1Ac+ donor cells in the peripheral blood at POD21 as compare to untreated rats (8.0 ± 3.2%, 6.2 ± 0.8% and 9.7 ± 1.1% vs 1.6 ± 1.2% of white blood cells, respectively). More specifically, we observed an increased frequency of donor granulocytes in all the groups treated with rapamycin and of monocytes in rats treated with Rapa-ISFI while no significant difference was observed in the frequency of donor T helper, T cytotoxic or B cells. In surviving rats, the percentage of donor leukocytes slightly decreased after POD21 but donor leukocytes were detectable until the endpoint (Fig. 5B). Interestingly, in Groups 2 and 3, the frequency of donor granulocytes and monocytes decreased to undetectable levels, whereas the frequency of donor T helper and cytotoxic cells increased over time until the endpoint. The two long-term survival recipients of Group 4 showed a stable level of chimerism with only a decreased frequency of donor granulocytes. As shown in Fig. 5C, a significant correlation was found between graft-survival and the frequencies of donor leukocytes (r = 0.51, p = 0.04), in particular granulocytes (r = 0.61, p = 0.01) and monocytes (r = 0.65, p = 0.006), measured on POD21.

We analyzed the donor and recipient vascularized bone marrow composition at the end point to verify potential changes induced by Rapa-ISFI treatment. We did not find any difference in the number of CD45^+^, B cells, T cells, monocytes and granulocytes or CD45^−^, and CD45^+^CD31^+^ cells of the contralateral or transplanted tibial bone marrow in the four groups (Supplementary Fig. S7). Notably, in transplanted (i.e., donor) bone marrow only a small number of donor cells were detectable, and most of the cells where of recipient origin. Furthermore, the number of bone marrow stem cells identified by the expression of CD34 and/or CD133 did not change among the groups and bone marrow origin (i.e., contralateral or transplanted) (Supplementary Fig. S8). Interestingly, a small number of donor stem cells were detected in contralateral and transplanted bone marrow in long-term surviving rats (POD100) while were not detectable in control, untreated rats.

### Induction therapy is needed to achieve high chimerism levels and long-term VCA survival

**Figure 6:**
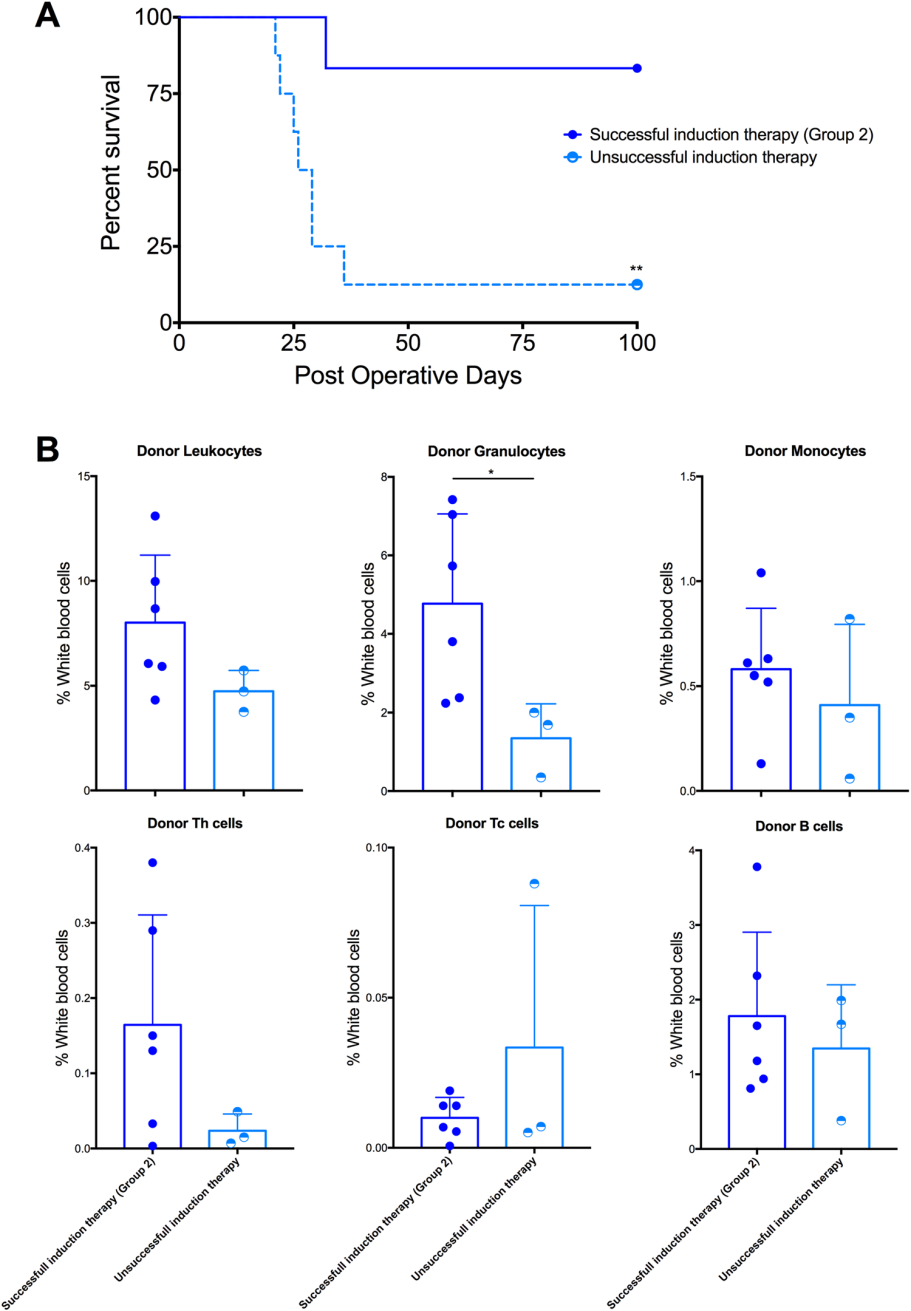
Induction therapy with anti-lymphocyte serum (ALS) is necessary for Rapa-ISFI promotion of allograft survival and for efficient induction of multilineage chimerism. (**A**) Survival of hind limb allografts in Rapa-ISFI treated rats with unsuccessful ALS therapy. Kaplan-Meier survival curves comparing allograft survival in recipient rats with unsuccessful induction therapy (*i*.*e*., white blood counts on the day of transplantation >7500 cells/µL) treated with ipsilateral injection of Rapa-ISFI as compared to rats of Group 2 (white blood counts on the day of transplantation <2500 cells/µL, from Fig. 2). **P < 0.01 by Mantel-Cox test. (**B**) Chimerism levels in rats with unsuccessful ALS therapy at POD21. The numbers of donor leukocytes, granulocytes and monocytes were measured in recipients with unsuccessful ALS therapy and compared to chimerism levels in rats of Group 2 (from Fig. 5A). Data presented as mean and SD, *P < 0.05 by unpaired T-test.

In order to understand the importance of ALS induction therapy to promote long-term VCA survival with Rapa-ISFI, in a new set of experiments we performed hind limb transplantation in recipients with unsuccessful ALS induction therapy. Rats were treated with bridging therapy and injection of Rapa-ISFI on the transplanted side at POD7 as described for Group 2. In rats with unsuccessful induction therapy, graft MST was 27.5 days, which is significantly shorter than rats of Group 2 (*i*.*e*., MST > 100 days, p = 0.007) (Fig. 6A). Moreover, the levels of multilineage chimerism in the blood were lower at POD21, with a significant reduction in the frequency of donor granulocytes (Fig. 6B).

### Rapa-ISFI treatment promotes the expansion of T regulatory cells

**Figure 7:**
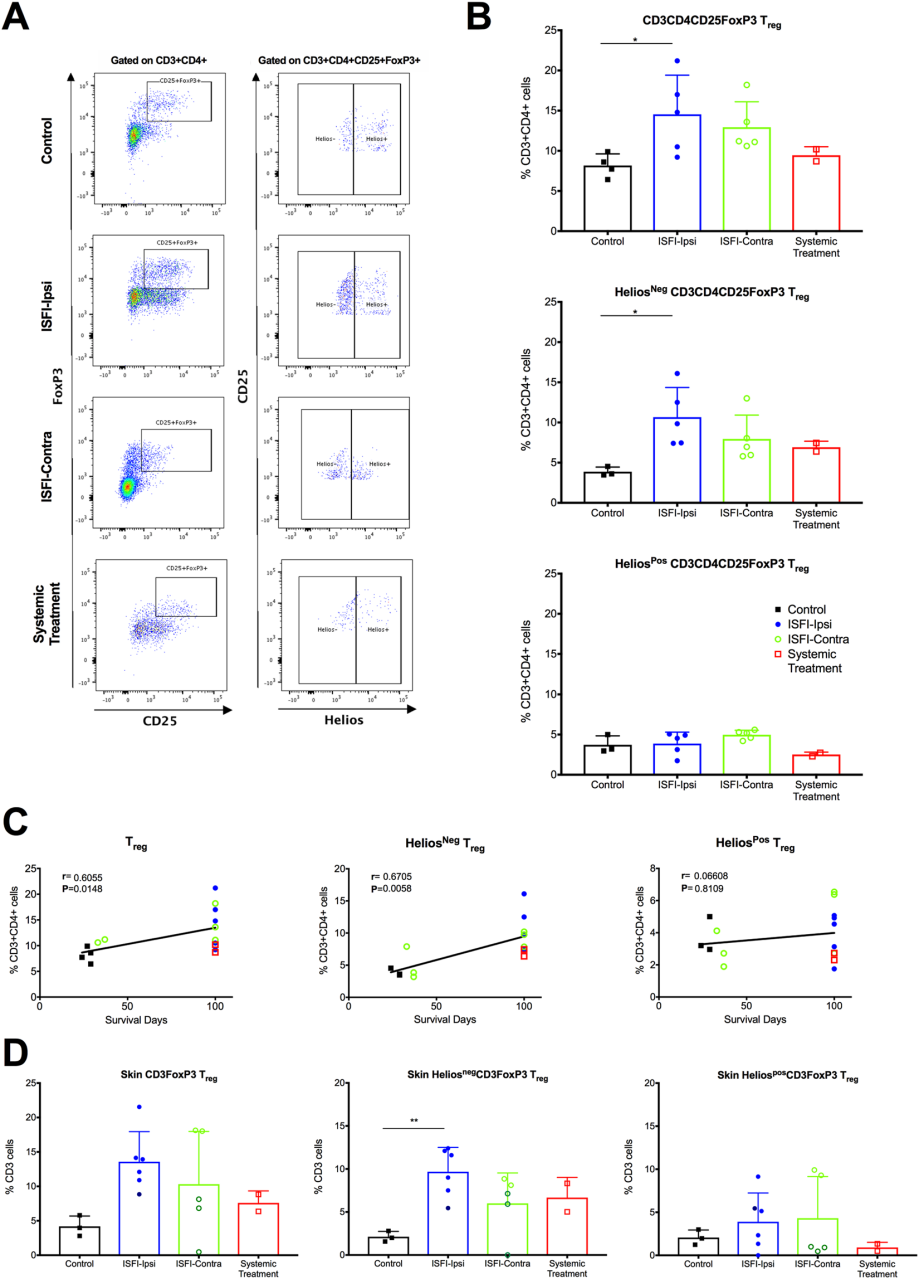
Rapa-ISFI injection on the transplanted side promotes expansion of blood and tissue T_reg._ (**A**) Representative flow cytometry for the quantification of T ^reg^, Helios^Neg^T_reg_ and Helios^Pos^T_reg_ in the peripheral blood at POD21. T_reg_ were identified as CD3^+^CD4^+^CD25^+^FoxP3^+^ cells. Helios^Pos^T_reg_ and Helios^Neg^T_reg_ were identified based on the expression of the transcription factor Helios. (**B**) Quantitative summaries for the frequency of T_reg_, Helios^Neg^T_reg_ and Helios^Pos^T_reg_ in the peripheral blood at POD21 (representative gating strategy presented in Supplementary Fig. 7). Data were expressed as frequency of CD3^+^CD4^+^ T cells and presented as mean and SD, *P < 0.05 by one-way ANOVA with Tukey’s multi-comparisons test. (**C**) Correlation analysis between T_reg_ frequencies and allograft survival. Frequency of T_reg_, Helios^Neg^T_reg_ and Helios^Pos^T_reg_ in peripheral blood of rats from Group 1 (closed squares), Group 2 (closed circles), Group 3 (open circles) and Group 4 (open squares) at POD21 were correlated with allograft survival by nonparametric (Spearman) correlation, r values and P values are reported for each correlation. (**D**) Frequency of T_reg_, Helios^Neg^T_reg_ and Helios^Pos^T_reg_ in skin recovered from the allograft the day of sacrifice. Skin samples were recovered and analyzed both from rats rejecting their grafts at sacrifice (darker dots) and long-term survivors at the endpoint. T_reg_ were identified as FoxP3^+^ cells after exclusion of doublets and selection of viable CD3^+^ cells. Helios^Neg^ and Helios^Pos^ cells were identified based on the expression of the transcription factor Helios. Data were expressed as frequency of CD3^+^ cells and presented as mean and SD, *P < 0.05, **P < 0.01 by one-way ANOVA with Tukey’s multi-comparisons test.

The frequency of circulating T_reg_ (CD3^+^CD4^+^CD25^+^FoxP3^+^), Helios^Pos^ and Helios^Neg^ T_reg_ was analyzed in the peripheral blood starting from POD21 (see Supplementary Fig. S9 for a representative gating strategy). As shown in Fig. 7A, when compared to Group 1, rats of Group 2 had significantly higher frequency of T_reg_ in the peripheral blood (p = 0.044), rats of Group 3 also had higher frequency of T_reg_ but it did not reach statistical significance (p = 0.145) and rats of Group 4 had unchanged T_reg_ frequency. Notably, the injection of Rapa-ISFI on the transplanted side promoted the expansion of Helios^Neg^T_reg_, without affecting the frequency of Helios^Pos^T_reg_. Correlation analysis between the frequency of T_reg_ at POD21 and graft survival time showed a significant correlation (r = 0.71, p = 0.006). Specifically, the survival time correlated with the frequency of Helios^Neg^ T_reg_ (r = 0.59, p = 0.001) and not with the frequency of Helios^pos^T_reg_ (Fig. 7B). The frequency of T_reg_ did not change significantly during the study and at POD100 the frequency of Helios^Neg^T_reg_ was similar to that at POD21 (see Supplementary Fig. S10).

At the time of rejection, the frequency of T_reg_ was analyzed in VCA skin of all rats after tissue digestion (see Supplementary Fig. S11 for a representative gating strategy). As shown in Fig. 7C, rats from Group 2 showed a significant increase in the frequency of Helios^Neg^T_reg_ in the transplanted skin as compared to untreated rats (9.68 ± 2.8 vs 2.12 ± 0.6%, respectively; p = 0.007). Notably, rats that rejected their grafts before the endpoint in Groups 2 and 3 presented lower frequency of Helios^Neg^T_reg_ in the skin (frequency was 5.45% in the rejecting rat of Group 2 and 4.35 ± 3.82% in the three rejecting rats of Group 3).

### Donor stimulation expands Treg in Rapa-ISFI treated rats *in vitro*

**Figure 8:**
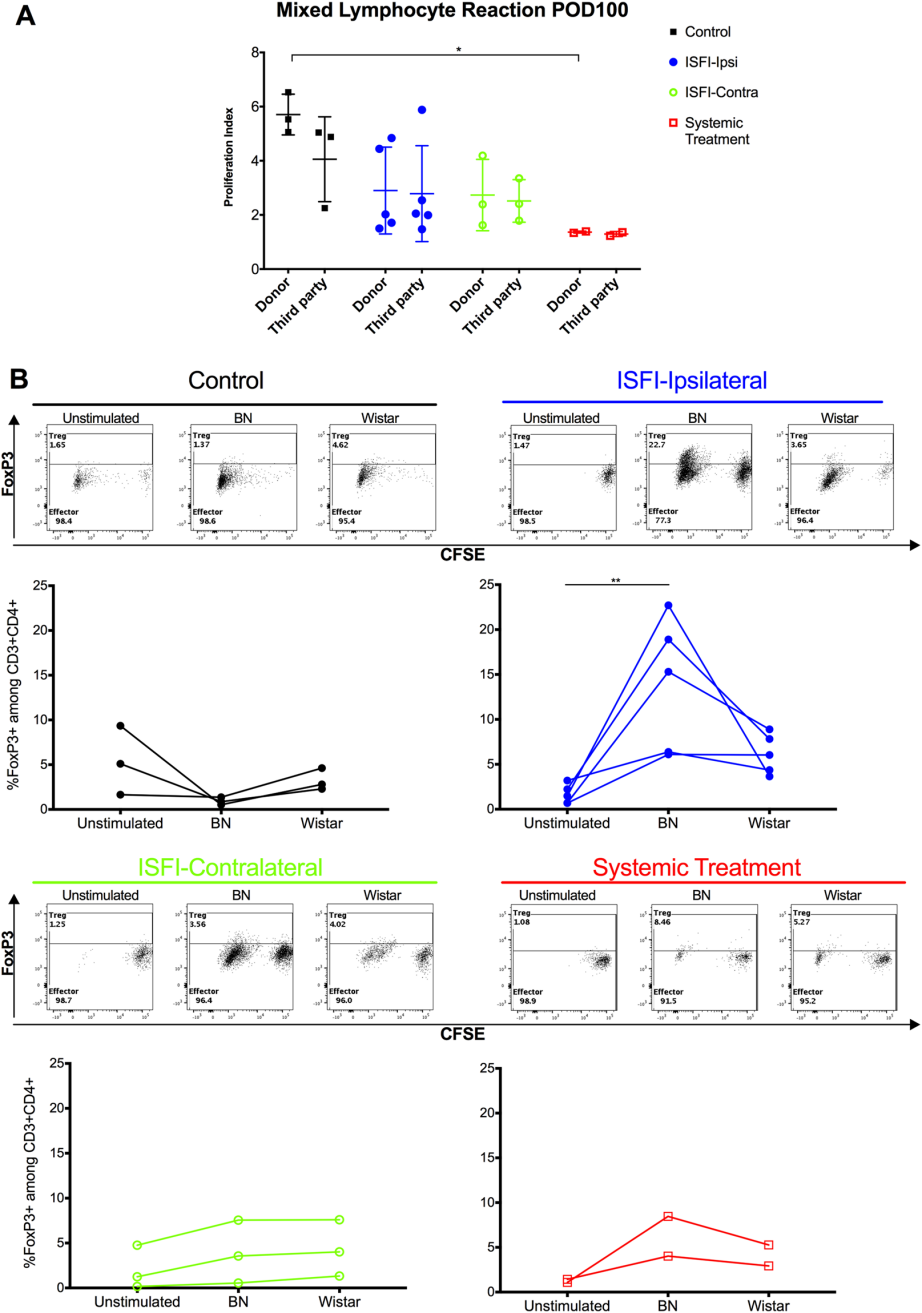
*In vitro* donor-specific stimulation induces T_reg_ in ipsilateral Rapa-ISFI treated rats. Mixed lymphocyte reaction (MLR) was performed using responder PBMC isolated at POD100 from long term surviving rats and compared to the response in PBMC isolated at rejection of untreated rats. Responder cells were mixed with gamma-irradiated stimulator cells isolated from spleens of Brown Norway (BN) donor rats or third party Wistar rats. Responder cells without stimulation (unstimulated control) were used as control of basal cell proliferation. After 5 days, cells were stained and analyzed by flow cytometry. (**A**) Untreated rats (Group 1, PBMC from 3 out of the 6 rats rejecting at POD 24–29), rats treated with Rapa-ISFI ipsilaterally (Group 2, from all the 5 surviving rats at POD100), contralaterally (Group 3, from all the 3 surviving rats at POD100) or with daily systemic rapamycin (Group 4, in the 2 surviving rats that did not show any signs of GvHD at POD 100) show comparable proliferation response to donor and third party stimulation. *P < 0.05 by one-way ANOVA with Tukey’s multi-comparisons test comparing the response either to BN or Wistar among the four groups. (**B**) Representative flow cytometry picture and quantification of T_reg_ induction in response to donor BN or third party (Wistar) stimulation in MLR culture of control, ipsilateral or contralateral Rapa-ISFI treated rats and systemically treated rats. Each dot represents the frequency of CD3^+^CD4^+^FoxP3^+^ T_reg_ in MLR of PBMC isolated from each rat stimulated with donor (BN) or third party (Wistar) cells. **P < 0.01 by one-way ANOVA with Dunnett’s multiple comparisons test comparing BN or Wistar stimulation to unstimulated control.

In order to assess the induction of donor specific tolerance, PBMC were isolated at POD100 and the T cell proliferative response to donor or third-party antigens was assessed *in vitro* by MLR and compared to the response in untreated rats (Group 1) at rejection. In the untreated rats of Group 1, donor (Brown Norway) and third party (Wistar) stimulations induced a strong proliferative response of CD4^+^ T lymphocytes in PBMC isolated at rejection (PI = 5.7 ± 0.8 and 4.1 ± 1.6, respectively). In rats of Groups 2 and 3, proliferation in response to donor and third-party stimulations was lower, but not significantly different as compared to rats from Group 1 (2.9 ± 1.6 and 2.8 ± 1.8 in Group 1 and 2.7 ± 1.3 and 2.5 ± 0.8 in Group 2 for donor and third party stimulation, respectively, Fig. 8A). In the two surviving rats from group 4, treated with daily systemic injection of rapamycin, a significant reduction of the proliferation was observed as compared to Group 1, both in response to donor or third party stimulation (PI = 1.4 ± 0.04 and 1.3 ± 0.1, respectively), suggesting a general hyporesponsiveness due to systemic immunosuppression.

When we analyzed the frequency of CD4^+^ T cells expressing FoxP3 in response to donor and third party stimulation we did not observe T_reg_ expansion in PBMC isolated from surviving rats from Group 1, 3 or 4. However, in rats receiving Rapa-ISFI ipsilaterally (Group 2) the percentage of T_reg_ significantly increased in response to donor antigens as compared to unstimulated controls while this frequency did not change in response to third party stimulation (14.2 ± 7.7%, 1.7 ± 1.1% and 6.2 ± 2.3%, respectively, Fig. 8B). The majority of the CD3^+^CD4^+^FoxP3^+^ cells were seen in the proliferating fractions.

## Discussion

In this study, we investigated a novel approach to immunosuppression regimens after transplantation. We used a biodegradable ISFI loaded with the immunoregulatory drug rapamycin to deliver low-dose immunosuppression and promote acceptance of VCA grafts by immunoregulatory mechanisms. Rapa-ISFI forms a drug depot that gradually releases rapamycin both *in vitro* and *in vivo*. The implant is simple to apply *via* subcutaneous injection and extends delivery times with systemic levels of the drug of about 30 ng/mL for the first 24 h and subtherapeutic systemic levels for up to 50–60 days. Compared to other drug delivery vehicles such as nanoparticles, microspheres, liposomes or hydrogels, the ISFI is easily accessible and allows for easy removal in case of development of complications that require the suspension/refinement of immunosuppressive therapy such as overwhelming infections, malignancies or adverse effects secondary to mTOR inhibition^25^.

When used in a VCA model, the ipsilateral injection of Rapa-ISFI promoted graft survival for >100 days. This consistent with the results achieved by other drug delivery systems such as FK506-loaded hydrogels^6,7^ or biodegradable disks containing FK506-loaded double-walled microspheres^9^, confirming that *in situ* delivery of immunosuppressive drugs is a feasible and promising approach in VCA. However, in contrast to FK506 hydrogels, ISFI presented a limited burst release that remained in the clinically relevant range. Moreover, systemic concentrations reached subtherapeutic levels one week after injection and were measurable until POD58 whereas tissue levels were measurable until POD100, demonstrating a lower systemic drug exposure and higher tissue concentrations than FK506 disks^9^. We recently reported the formation of a capsule around injected tacrolimus-loaded hydrogel^26^ and the same is reported for other ISFI and biomaterials^27^. It is likely that also Rapa-ISFI are encapsulated after injection. However, we could not retrieve any of the implant in long term surviving rats, suggesting that, similarly to tacrolimus hydrogels^26^, the formation of a fibrous wall did not prevent the interaction of the ISFI with the surrounding tissues. However, the degree of the foreign body reaction triggered by the ISFI injection should be further investigated to support the translation of this approach to patients.

Aside from the development of innovative drug delivery systems for site-specific immunosuppression, the possibility to induce immunoregulatory mechanisms to modulate the host immune response to the engraftment (e.g. by promoting chimerism and/or expansion of regulatory cells) has been investigated as a potential solution for minimizing immunosuppression-related complications in VCA^28,29^. In this study we argue that these two strategies may be combined. Thanks to a wise selection of the drug, the delivery system and the injection site, we could deliver reduced but effective immunosuppression and promote immunoregulation preventing graft rejection.

We show that sustained low-dose delivery of rapamycin by Rapa-ISFI could promote significantly higher levels of chimerism of both lymphoid and myeloid lineages at POD21. At this time, the levels of myeloid chimerism were elevated and positively correlated with graft survival, suggesting that initial high levels of donor granulocytes and monocytes may correlate with the engraftment of donor pluripotent hematopoietic stem cells (HSC) as recently demonstrated in mice receiving HSC transplantation after antibody-mediated clearance of recipient HSC^30–32^. To support this hypothesis we performed bone marrow analysis demonstrating a the presence of a small number of donor-derived CD34^+^ and/or CD133^+^ cells residing in the recipient bone in all the non-rejecting rats from Groups 2, 3 and 4. Circulating donor T cell levels, although low at the beginning of treatment, increased with time, reaching the highest values at the endpoint in all rapamycin-treated groups, further confirming the capacity of rapamycin to promote engraftment of donor HSC and therefore graft survival^33^.

Additionally, our study clearly shows that low-dose rapamycin delivered by ISFI, induced T_reg_ cells in the peripheral blood and in VCA-skin. The capacity of rapamycin to induce T_reg_ alone or in combination with other treatments has been extensively reported^30–32^. We demonstrated that the frequency of T_reg_ in blood and VCA-skin correlated with the promotion of graft survival. Notably, Rapa-ISFI treatment specifically promoted the expansion of Helios^Neg^T_reg_, as recently reported in a nonhuman primate model of kidney transplantation with rapamycin-only treatment or in combination with anti-CD28 therapy^33^. It was previously proposed that Helios expression is restricted to thymus-derived natural T_reg_ (nT_reg_) distinguishing them from peripheral T_reg_ (pT_reg_)^34^. Therefore, the accumulation of Helios^Neg^T_reg_ can be seen as a direct expansion of pT_reg_. In line with this idea, the majority of the circulating and skin-resident T_reg_ were Helios^Neg^ and the majority of thymus T_reg_ were Helios^Pos^ in our model (unpublished observation), confirming a good correlation of this marker with pT_reg_. However, recent studies excluded the value of Helios as a marker of nT_reg_ and proposed that Helios^Neg^T_reg_ may have an unstable but normal suppressive function^35–38^. We believe that the two hypotheses are not mutually exclusive and that Rapa-ISFI can promote the generation of Helios^Neg^ pT_reg_ with reduced stability, which accumulate in VCA-skin and consequently contribute to graft acceptance. Accordingly, in the MLR experiment we observed a proliferating response to donor stimulation but a significant expansion of donor specific T_reg_ in rats of Group 2, similar to what has been reported in rapamycin-treated human MLR^39^. Conversely, in Group 4, a general hyporesponsiveness to stimulation due to systemic immunosuppression was observed- This confirms the hypothesis that *in situ* delivery of rapamycin contributes only minimally to systemic immunosuppression. The stability and the importance of these peripheral immunoregulatory mechanisms, mediated by Helios^Neg^T_reg_, remains unclear and deserves further investigation, especially after infection or by-stander activation^40,41^.

Interestingly, lethal GvHD occurred in 60% of the rats treated with systemic rapamycin although systemic drug levels remained within the recommended therapeutic window (*i*.*e*. 10–20 ng/mL for regimens without calcineurin inhibitors^42^). Development of GvHD is rare after VCA and has been reported mainly after recipient irradiation and bone marrow transplantation^43^. Notably, rats treated with systemic rapamycin showed the highest levels of mixed chimerism, while unsuccessful induction therapy was associated with lower chimerism levels and lower graft survival. This suggests that a combination of systemic application of rapamycin with immunodepletive agents may promote an excessive engraftment of donor cells and thus development of GvHD. Conversely, Rapa-ISFI treatment did not induce GvHD, likely due to the lower rapamycin dose and lower systemic levels. Therefore, site-specific delivery of mTOR inhibitors may promote a better balance between multilineage mixed chimerism and GvHD development. Accordingly, the contralateral injection of Rapa-ISFI also prolonged graft-survival and induced multi-lineage chimerism without any signs of GvHD. However, as compared to injection at the transplanted site, contralateral Rapa-ISFI injection was less efficient in terms of induction of pT_reg,_ resembling the response of systemic rapamycin treatment. This suggests that the injection on the ipsilateral side may promote stronger immunoregulation due to the co-presence of rapamycin and abundant donor-antigens, especially in the draining lymph nodes. This may shift the lymph node and local microenvironment towards a regulatory function, driving donor-specific peripheral tolerance. Although we did not specifically looked at lymph node response in this study, this hypothesis is supported by recent findings clearly showing that co-delivery of the antigen with rapamycin can be used to induce antigen-specific immunological tolerance in peripheral lymph nodes^44,45^. Further studies will be necessary to definitively prove this hypothesis and to better characterize lymph node response that remains unexplored in the current study.

Although mTOR inhibition offer the advantage of low nephrotoxicity, lower incidences of viral infection and beneficial effects on endothelial cell proliferation, various adverse events have been reported in transplant patients using mTOR based immunosuppression^25,46^. These include (but are not limited to) hematological complications (i.e., thrombocytopenia, leukopenia, neutropenia, lymphopenia) and anaemia, insulin resistance and diabetes, glomerular dysfunction and renal failure, dyslipidemia, mucositis, pneumonitis, lymphedema, angioedema and osteonecrosis^25,46^. However, we believe that local, low-dose rapamycin therapy with Rapa-ISFI will help to prevent the development or reduce the incidence of adverse events as compared to systemic immunosuppression, similarly to what was recently reported for tacrolimus-loaded hydrogels and other drug-delivery system^7,47^. Moreover, the explanation of the Rapa-ISFI, as discussed above, may offer an easy approach to mTOR inhibitors discontinuation to promote the resolution of adverse events. Specifically designed studies are warranted to definitively prove the potential of localized, low-dose rapamycin to mitigate immunosuppression-related morbidities in transplant recipients.

The main limitation of this study is the lack of functional proof that the expansion of T_reg_ and the increased chimerism levels may directly control alloreaction promoting graft survival. It is a well-established paradigm that a balance of T_reg_ over T effector cells determines immune tolerance in transplantation^11,12^. Similarly, it is clear that establishment of chimerism, even transient, can lead to tolerance induction in animal models and kidney-transplanted patients. An important question posed by our experiment is whether the Rapa-ISFI induced pT_reg,_ would be able to specifically inhibit alloreaction. We did not specifically address this question, however the literature reports that T_reg_ isolated from rapamycin-treated MLR specifically inhibited newly prepared MLR assays and concurrently recruited more autologous responder T_reg_^39,48^. Similarly, T_reg_ accumulating in the periphery of long-term survivors with self-resolving acute rejection episodes receiving IL2 fusion protein showed donor-specific suppression *in vitro*^49^. Therefore, we believe that Rapa-ISFI-induced donor-specific pT_reg_ may indeed control allo-response in the periphery. However, more studies are necessary to specifically address the role of the newly generated T_reg_ in the control of VCA rejection. In particular, it would be important to verify what would happen after the 100 days endpoint of this study. Is a second injection of Rapa-ISFI necessary to prolong graft survival as suggested for tacrolimus hydrogels^7^, or the immunoregulatory mechanisms would be sufficient to avoid graft rejection? Considering the lack of efficacy of tolerogenic protocols reported in the clinic^50^, it would be important to discern the role of immunoregulation from the one played by low-dose drug concentration in a clinically relevant animal model. We speculate that the immunoregulatory mechanisms alone would be not sufficient for VCA survival in large animal models and human patients but they may co-operate with low-dose immunosuppression guaranteed by multiple injection of Rapa-ISFI to promote long-term survival in a clinical setting. We will investigate this hypothesis in a porcine VCA model. Another limitation of the study is the lack of a secondary donor skin graft to better assess the capacity of the pT_reg_ in combination with low-dose rapamycin to inhibit donor-specific response to a secondary *in vivo* challenge. The normal T cell proliferating response in the MLR assay and the observation of rejection episodes in Rapa-ISFI-treated rats suggest that the secondary challenge would be likely rejected, also due to by-stander activation secondary to surgical trauma. However, the recently proposed possibility that “memory of regulation” can dominate over memory of “infection-triggered rejection^41^” deserves further verification, and it will be explored in additional studies.

In conclusion, in this study we have developed a new therapeutic protocol combining induction regimens and regional delivery of rapamycin by ISFI. We showed that local drug delivery of immunosuppressive drugs could be used not only to promote less toxic immunosuppressive protocols increasing patient compliance, but also to favor the reprogramming of the local response towards a regulatory function. Moreover, we provide evidence that delivery of rapamycin using an ISFI promotes immunoregulatory mechanisms such as establishment of multilineage chimerism and donor-specific pT_reg_, which may mitigate the response to the graft resulting in long-term VCA survival.

## Materials and Methods

### Preparation and evaluation of rapamycin-loaded *in situ* forming implant (ISFI)

We developed a rapamycin-loaded *in situ* forming implant (Rapa-ISFI) analogous to the Atrigel^®^ delivery system for long-term regional release^51,52^. To this aim, rapamycin (5 mg, LC Laboratories, Woburn, MA, USA) was dissolved in 0.31 mL N-methyl-2-pyrrolidone (NMP, Sigma-Aldrich Chemie GmbH, Buchs, Switzerland) prior to being added to poly(D,L-lactic-co-glycolic acid) (PLGA, Resomer^®^ RG 502, 50:50 mol% lactide/glycolide, 7–17 kDa, Sigma-Aldrich) at a final concentration of 45% (w/v) PLGA. The resulting viscous ISFI was then transferred into a 1 mL syringe and injected within 24 h. The release kinetics of the implant were evaluated *in vitro* and *in vivo* (detailed information is provided in the *Supplementary material)*.

### Animal experiments

Inbred Lewis (recipient) and Brown Norway (donor) rats (all male) weighing between 200 g and 250 g were purchased from Charles River (Sulzfeld, Germany). All animals were housed in Specific Pathogen Free (SPF) conditions in cages of 2–4 rats with water and food ad lib. Animal experiments were performed in accordance with the terms of the Swiss animal protection law and were approved by the Animal Experimentation Committee of the Canton of Bern, Switzerland. Experimental protocols were refined according to the 3 R principles and state-of-the-art anesthesia and pain management were used to minimize the number of animals and to reduce the exposure of the animals to stress and pain during the experiments.

### Experimental design

To evaluate the clinical efficacy of the therapy in a VCA model, Brown Norway-to-Lewis hind limb transplantations were performed as described previously with modifications^6^. Anti-lymphocyte serum (ALS), 0.5 mL/rat was injected intraperitoneally 4 days before and 1 day after transplantation. The success of the ALS induction therapy was monitored by measuring the number of leukocytes in the peripheral blood on the day of transplantation. Rats with a leukocyte count lower than 2500 cells/µL of blood were used as recipients of hind limb transplants. After hind limb transplantation, animals were treated with FK506 for 6 days (0.5 mg/kg subcutaneously) to bridge the time until complete wound healing to prevent impaired healing caused by rapamycin^53,54^. On day 7, animals were divided into 4 groups: Group 1 was left untreated (Control, n = 6); Group 2 received an ISFI loaded with 5 mg of rapamycin subcutaneously into the groin of the transplanted limb (ISFI-Ipsilateral, n = 6) and Group 3 into the groin of the contralateral limb (ISFI-Contralateral, n = 6) (see Supplementary Fig. 1). Group 4 received daily injections of 0.5 mg/kg rapamycin subcutaneously (Systemic treatment, n = 5). Clinical rejection was graded macroscopically and rats were sacrificed either once grade 3 rejection was reached or on day 100, which was defined as the endpoint. Rapamycin levels were measured in 1) blood at different time points, 2) skin-biopsies retrieved from the graft on postoperative day (POD) 21 and 49 and 3) skin, fat pad, muscle of the graft and of the contralateral side at the end of the experiment. To analyze the importance of ALS therapy in the therapeutic protocol, eight Lewis rats with unsuccessful ALS depletion (*i*.*e*., blood leukocyte count higher than 7500 cells/µL, after the first ALS injection) underwent hind limb transplantation and were treated as described for Group 2 (*i*.*e*., FK506 bridge therapy, Rapa-ISFI-ipsilaterally on day 7). Graft-versus-host disease (GvHD) was assessed macroscopically, by mixed lymphocyte reaction (MLR), and by analyzing the number and origin of infiltrating lymphocytes into injured sites by flow cytometry. Detailed material and methods are available in the Supplementary Information.

### Flow cytometry analysis of chimerism and T regulatory cells

EDTA-2K whole blood was collected and red cells were lysed using erythrocyte lysis buffer (eBioscience, Vienna, Austria). Cells were then stained with Fixable Viability Dye eFluor 506 (eBioscience), washed and incubated with anti-rat fluorochrome-conjugated antibodies against CD3, CD8 (Miltenyi Biotec GmbH, Bergisch Gladbach, Germany), CD4 and CD25 (eBioscience) or the Brown Norway specific marker RT1Ac (MHC Class I, clone MCA 156/OX-27, AbD Serotec, Kidlington, UK). For T_reg_ staining, cells were fixed after extracellular staining and permeabilized using the FoxP3/Transcription Factor Staining Buffer Set (eBioscience) and incubated with anti-FoxP3 (eBioscience) and anti-Helios (Miltenyi) antibodies. After washing, cells were analyzed by flow cytometry using a SORP LSRII flow cytometer (BD Biosciences, San Diego, CA, USA) and BD Diva Software. Data were analyzed using FlowJo software (Tree Star, Ashland, OR, USA). Positivity for the RT1Ac marker was determined using cells from naïve Lewis rats as negative controls. Fluorescence minus one (FMO) controls were used to set the cut-off for the T_reg_ analysis. Skin (dermal and epidermal tissue), ear and tongue were collected and subcutaneous fat and hairs were carefully removed. Tissue was thoroughly minced into small pieces and incubated with agitation in DMEM (Thermo Fisher Scientific, Waltham, MA, USA) containing 10% FBS (Thermo Fisher) and 1 mg/mL Dispase (StemCell Technology, Vancuver, Canada) overnight at 4 °C. Tissue was washed with DMEM medium (Thermo Fisher) and digested with DMEM containing 10% FBS, 1 mg/mL Collagenase D (Roche, Basel, Switzerland) and 200 μg DNase I (Sigma-Aldrich) for 1 h at 37 °C in agitation. After filtration of the resulting single cell suspension through 40 μm cell strainers, cells were washed and mononuclear cells isolated using Ficoll Separation Media (GE Healthcare, Europe GmbH, Switzerland). Isolated cells were processed for flow cytometry as described above. CD4 and CD8 expression was partially lost due to the digestion step. Therefore, in the tissue, T_reg_ cells were identified as CD3^+^FoxP3^+^ cells.

### Mixed lymphocyte reaction

MLR was performed as previously described with minor modifications^55^. Briefly, responder cells were isolated from peripheral blood of long-term survival animals, frozen and stored at −150 °C until analysis. After cell thawing, peripheral blood mononuclear cells (PBMC) were stained with 5 µM carboxyfluorescein succinimidyl ester (CFSE, Thermo Fisher). Stimulator cells were isolated from spleens of donor (Brown Norway) or third party rats (Wistar) using gentle sieving/mincing methods. Cells were then frozen and stored until needed and stimulator cell proliferation blocked using 30Gy gamma-irradiation. After extensive washing, responder and stimulator cells were mixed in a 1:1 ratio and incubated for 5 days in DMEM, 10% FBS, 1% PenStrep (Thermo Fisher) and 0.05 mM 2-mercaptoethanol (Sigma-Aldrich). Responder cells without stimulation (unstimulated control) were used as the control of basal cell proliferation. After 5 days, cells were stained for T_reg_ as described before and analyzed by flow cytometry. Proliferation index (PI) was determined using FlowJo software.

### Statistical Analysis

Statistical analysis was performed using the GraphPad Prism version 7. Unless noted otherwise, the results are expressed as means ± SD. Survival of the allografts was examined using Kaplan-Meier analysis, and groups were compared using the log-rank test. Two-tailed *t* test was used to compare two groups, one-way ANOVA with Tukey’s multiple comparisons test was used to compare means of more than 2 groups and one-way ANOVA with Dunnett’s multiple comparisons unpaired test was used to compare treated groups (Groups 2–4) to the untreated group (Group 1). Paired or unpaired tests were used when appropriate as reported in the figure legend. Correlation was measured using Spearman’s (rank) correlation. Significance was defined as p < 0.05. Rats developing lethal GvHD in Group 4, were excluded from the chimerism and T_reg_ analysis. Moreover, considering that some sample was lost due to technical problem during sample preparation, N number and scatter plot were show for all the figures. Significance was defined as p < 0.05.

## Supplementary information

Supplementary Information
