# Supplementary material for: Delivery of Rapamycin Using In Situ Forming Implants Promotes Immunoregulation and Vascularized Composite Allograft Survival
Source: Sci Rep. 2019 Jun 25;9:9269. doi: 10.1038/s41598-019-45759-y (PMC6592945; doi:10.1038/s41598-019-45759-y)
Supplement: Supplementary file 1 — Supplementary Information [file 41598_2019_45759_MOESM1_ESM.docx]

Supplementary Information

Delivery of Rapamycin Using *In Situ* Forming Implants Promotes Immunoregulation and Vascularized Composite Allograft Survival.

Damian Sutter, Dzhuliya V. Dzhonova, Jean-Christophe Prost, Cedric Bovet, Yara Banz, Lisa Rahnfeld, Jean-Christophe Leroux, Robert Rieben, Esther Vögelin, Jan A. Plock^*^, Paola Luciani^*^, Adriano Taddeo^*^, Jonas T. Schnider^*^

*** Correspondence:** Corresponding Authors: [esther.voegelin@insel.ch](mailto:Esther.Voegelin@insel.ch) - [jan.plock@usz.ch](mailto:jan.plock@usz.ch) - [paola.luciani@uni-jena.de](mailto:paola.luciani@uni-jena.de) - [adriano.taddeo@dbmr.unibe.ch](mailto:adriano.taddeo@dbmr.unibe.ch)

# Supplementary Methods

## In vitro analysis of ISFI release pattern

A release medium composed of, 0.9% sodium chloride (Sigma-Aldrich Chemie GmbH, Buchs, Switzerland), 0.5% polysorbate 80 (Carl Roth GmbH, Karsruhe, Germany) and 10% methanol (Carl Roth) was prepared ad 1 L water. Stainless steel mesh baskets (1) were suspended in 50 mL release medium and stored under horizontal shaking in an incubator at 37 °C. The prepared rapamycin-loaded ISFI (Rapa-ISFI) was injected into the baskets. Release medium was replaced with fresh medium at regular time intervals and aliquots of the release medium were sampled, lyophilized and stored at -20 °C. Samples were taken over the course of 28 days. At the end of the experiment, the lyophilized samples were reconstituted with methanol and tacrolimus 25 µg/mL as an internal standard to quantify rapamycin using high-performance liquid chromatography (1260 Infinity, Agilent, Santa Clara, CA, United States). A YMC-ODS AQ column (150x4.6mm ID, 3um particle size, YMC Co Ltd., Kyoto, Japan) was preheated to 50°C. The mobile phase consisted of 1mL/min 20% ultrapurified water, 50% methanol and 30% acetonitrile, set up as an isocratic method. Readout was measured at 278 nm and analyzed using Agilent ChemStation Software (Agilent).

## Evaluation of the systemic concentration of rapamycin in naive animals after ISFI injection

Rapa-ISFI was injected subcutaneously in the groin area of three naïve Lewis rats. 0.2-0.5 mL of whole blood was collected from the tongue vein at designated time points and rapamycin levels were measured by LC-MS/MS (see below for details).

## Animal experiments

Inbred Lewis (recipient) and Brown Norway (donor) rats (all male) weighing between 200 g and 250 g were purchased from Charles River (Sulzfeld, Germany). All animals were housed in Specific Pathogen Free (SPF) conditions in cages of 2-4 rats with water and food ad lib. Animal experiments were performed in accordance with the terms of the Swiss animal protection law and were approved by the Animal Experimentation Committee of the Canton of Bern, Switzerland. Experimental protocols were refined according to the 3R principles and state-of-the-art anesthesia and pain management were used to minimize the number of animals and to reduce the exposure of the animals to stress and pain during the experiments.

## Surgical procedure for hind limb transplantation

Rat hind limb transplantation was performed as described previously with several modifications (2,3). Briefly, buprenorphine at 50 mg/kg was given as a preemptive analgesic followed by 5% isoflurane in pure oxygen inhalation anesthesia for the induction and 1.5% for maintenance. Donors (Brown Norway) animals’ hindlimbs were amputated at the midfemur level. The donor’s inguinal fat pad was detached from the hind limb. Recipient (Lewis) rats were prepared by amputating the corresponding hindlimb leaving the autologous fat pad intact. Endomedullary osteosynthesis was performed with a blunted 18-gauge cannula. The femoral artery was anastomosed in an end-to-end technique with 10/0 interrupted sutures, and the vein anastomosis was performed with a cuff technique using a polyimide tube (RiverTech Medical, Chattanooga, TN USA) as described previously(4). After ensuring adequate vascularization of the transplanted limb, the femoral and sciatic nerves were anastomosed with interrupted 10/0 sutures followed by muscle and skin adaptation with 4/0 resorbable sutures (B. Braun Surgical, Rubi, Spain).

## Experimental group treatment

A power analysis was conducted to determine sample size using G*Power 3.1. A minimum difference of 35% in the mean frequency of FoxP3^+^ cells in CD4^+^CD25^+^ cells between a control group of rats and a treated group was considered of biological importance. The mean frequency and standard deviation of the FoxP3+ cells in CD4^+^CD25^+^ has been previously reported by our group (i.e. 44.8±8.8) (5). Therefore we calculated that a sample size of n=6 for each group would have the 80% power to detect difference between two independent means with an effect size d=1.784091, a significance level of 5%, and a two-sided test.

In order to improve precision, experimental conditions were applied in a smaller number of animals (1-3) and repeated till the necessary sample size was reached (i.e., transplantations were performed in 1-3 animals/day and animals were treated and allocated in a experimental group as described below). Therefore, each group was the result of the enrolment of rats coming from 3 “experimental blocks” performed in a timespan of about two years.

After hind limb transplantation, each animal (experimental unit) was independently randomized to the treatment conditions. All the recipients underwent an induction therapy with anti-lymphocyte serum (ALS) on day 4 preoperatively and postoperative day (POD) 1 (0.5 mL/rat i.p). Success of the induction therapy was measured right before operation (see below) and Brown Norway-to-Lewis hind limb transplantations were performed as described above. After hind limb transplantation, all animals were treated with 0.5 mg/kg FK506 subcutaneously in the neck starting at day 0 until day 6 to bridge the time to complete wound healing. On day 7, rats were randomly divided in 4 treatment groups: Group 1 was left untreated (Control, n=6); Group 2 received an ISFI loaded with 5 mg of rapamycin subcutaneously into the groin fat pad of the transplanted limb (ISFI-Ipsilateral, n=6); Group 3 received an ISFI loaded with 5 mg of rapamycin subcutaneously into the groin fat pad of the contralateral limb (ISFI-Contralateral); Group 4 received daily injections of 0.5 mg/Kg rapamycin subcutaneously (Systemic treatment, n=5). The treatment was independently applied to each animal and animals of different groups were hosted in the same cages. All animals were evaluated daily for general well-being and clinical rejection was graded macroscopically as 0=no rejection, 1=erythema and edema, 2=epidermolysis and exudation, and 3=desquamation, necrosis, and mummification. The rats were sacrificed either once grade 3 (rejection) was reached or at day 100 (end-point).

## Evaluation of the number of lymphocyte after anti-lymphocyte serum (ALS) injection

In order to evaluate the success of the ALS therapy, blood was collected from all ALS treated Lewis rats right before the transplantation in dipotassium ethylenediamine tetraacetic acid (K2EDTA, Sarstedt AG, Nümbrecht, Germany) and analyzed with a blood cell counter (Sysmex Suisse AG, Horgen, Switzerland) within 30 minutes of collection. Rats with a white blood cell count <2500 cells/µL were used as hind limb transplant recipients of Groups 1-4. Moreover, 8 rats with unsuccessful ALS depletion (i.e. with blood cell count >7500 cells/µL) were used to understand the importance of ALS induction therapy to promote long-term survival in ipisilaterally injected Rapa-ISFI treated rats.

## Histopathology

Tissue samples from the grafts, retrieved at the end of the experiments, were fixed in 4% buffered formaldehyde, processed according to standard histopathological specimen work-up, sectioned at 3 µm thickness and stained with hematoxylin and eosin (H&E) for microscopic evaluation. A pathologist blinded to treatment groups, scored all the samples. Graft rejection was evaluated in skin samples based on the Banff 2007 working classification of skin(6). Moreover, skin samples were analyzed for lymphocyte infiltration, vessels and endothelial cells pathology and tissue necrosis. For each of these categories a score from 0 to 3 was given (i.e., 0= absent, 1=minimal, 2=moderate or 3=extensive). For muscle histology a score from 0 to 3 (i.e., 0= absent, 1=minimal, 2=moderate or 3=extensive) was given for necrosis and lymphocyte infiltration, the sum of these two categories gave the final muscle histopathological score.

## Quantification of rapamycin in plasma and tissue

Whole blood samples were collected into tubes containing EDTA-2K at different time points and stored at –20°C until analysis. Rapamycin levels were measured by LC-MS/MS using the Kit MS1100 (ClinMass® Complete Kit, advanced, for Immunosuppressants in Whole Blood, RECIPE Chemicals + Instruments GmbH, Munich, Germany). The lower limit of quantification of rapamycin was 1.5 ng/mL.

Tissue levels were measured in skin biopsies retrieved from the transplant collected at POD21 and 49 as well as in skin, muscle, fat pad tissues, both from the transplanted and the contralateral sides. After tissue collection, 40 mg was aliquoted in a 2 mL Eppendorf tube and all samples were stored at -80°C. The sample preparation was adapted using the MS1312 from Recipe as internal standard (IS). Rapamycin and IS were dissolved in 70% (v/v) methanol solution. Standard spiking solution were prepared to build up a calibration curve between 25 to 750 ng/mL, and the QC concentrations were set at 35, 150 and 700 ng/mL. The frozen tissues were gently thawed at room temperature. For blank matrix, calibration and QCs samples tissue without rapamycin treatment was needed. To prepare the calibration curve and the QC samples, 40 µL of standard spiking solution (25 - 750 ng/mL), 40 µL of IS solution, 920 µL of precipitation solution (MS1021) were added to untreated tissue. A blank matrix is prepared adding 1000 µL of precipitation solution to untreated tissue. A volume of 40 µL of IS solution and 960 µL of precipitation solution were added to the treated samples. All samples were then grinded with five stainless steel balls for 30 minutes at 25 Hz. The tubes were centrifuged 5 minutes at 4°C and 20’000 rcf. 500 µL of the tissue extract was filtered with a Mini-Uni Prep G2 vials (GE Healthcare, Chicago, USA).

Chromatographic analysis was performed on an Acquity I-Class system (Waters, Milford, MA, USA) with ClinMass® Complete Kits (Immunosuppresants in Whole Blood, advanced – on-line analysis). The autosampler temperature was set at 10 °C and the autosampler needle was washed with a strong needle wash solution of isopropanol:methanol:acetontitrile:H2O (1:1:1:1, v/v). A solution of 20% (v/v) methanol was used as weak needle wash. Analytes were ionized by electrospray ionization (ESI) in the positive mode and detected on a triple quadrupole mass spectrometer (Xevo TQ-S, Waters, Milford, MA, USA). The capillary and the cone voltage were set at 3 kV and 40 V, respectively. The source offset was set at 60 V, the desolvation temperature at 400 °C, the desolvation gas at 1000 L/h, the cone gas at 150 L/h, the nebulizer at 7 bar and the source temperature at 150 °C. The transition parameters for each transition are summarized in Table 1.

| Name | Ion | Parent [m/z] | Daughter [m/z] | Collision [V] |
| --- | --- | --- | --- | --- |
| Sirolimus | Quantifier | 931.6 | 864.6 | 16 |
| Sirolimus 13C-d2 | Quantifier | 935.6 | 864.6 | 16 |
| Sirolimus | Qualifier | 931.6 | 846.6 | 19 |
| Sirolimus 13C-d2 | Qualifier | 935.6 | 846.6 | 19 |

**Table 1**: SRM parameters for Rapamycin and Rapamycin 13C-d2 quantifiers and qualifiers ions

The instrument was controlled via MassLynx (version 4.1, Waters). Data were acquired, integrated and processed with TargetLynx (MassLynx v4.1).

## Bone marrow characterization

At the end point one tibia from the contralateral side and one from the transplanted side (i.e., tibia from donor origin) were isolated for each rat. Bone was cut and the bone marrow was extensively flushed with PBS using a syringe and a 21G needle. After extensive resuspension, cells were filtered using a 70 µm mesh, frozen in 90% FBS and 10% DMSO and stored at 150 °C. For the staining cells were thawed, extensively washed with PBS/BSA 1% and resuspended in 500 µL. Cells were then stained with a solution of 0.5 µg/mL Hoechst 33342 dye for 20 minutes at 4 °C, washed and incubated with anti-rat fluorochrome-conjugated antibodies against CD3, CD45R (Miltenyi Biotec GmbH, Bergisch Gladbach, Germany), CD45, CD4 and CD31 (eBioscience), CD34 and CD133 (Novus Biologicals, Centennial, USA) and the Brown Norway specific marker RT1Ac (MHC Class I, clone MCA 156/OX-27, AbD Serotec, Kidlington, UK). After washing, cells were analyzed by flow cytometry using a SORP LSRII flow cytometer (BD Biosciences, San Diego, CA, USA) and BD Diva Software. Data were analyzed using FlowJo software (Tree Star, Ashland, OR, USA). Absolute number was calculated using Precision Count Beads (BioLegend) following the manufacturer instructions.

# Supplementary Figures

**A**

**
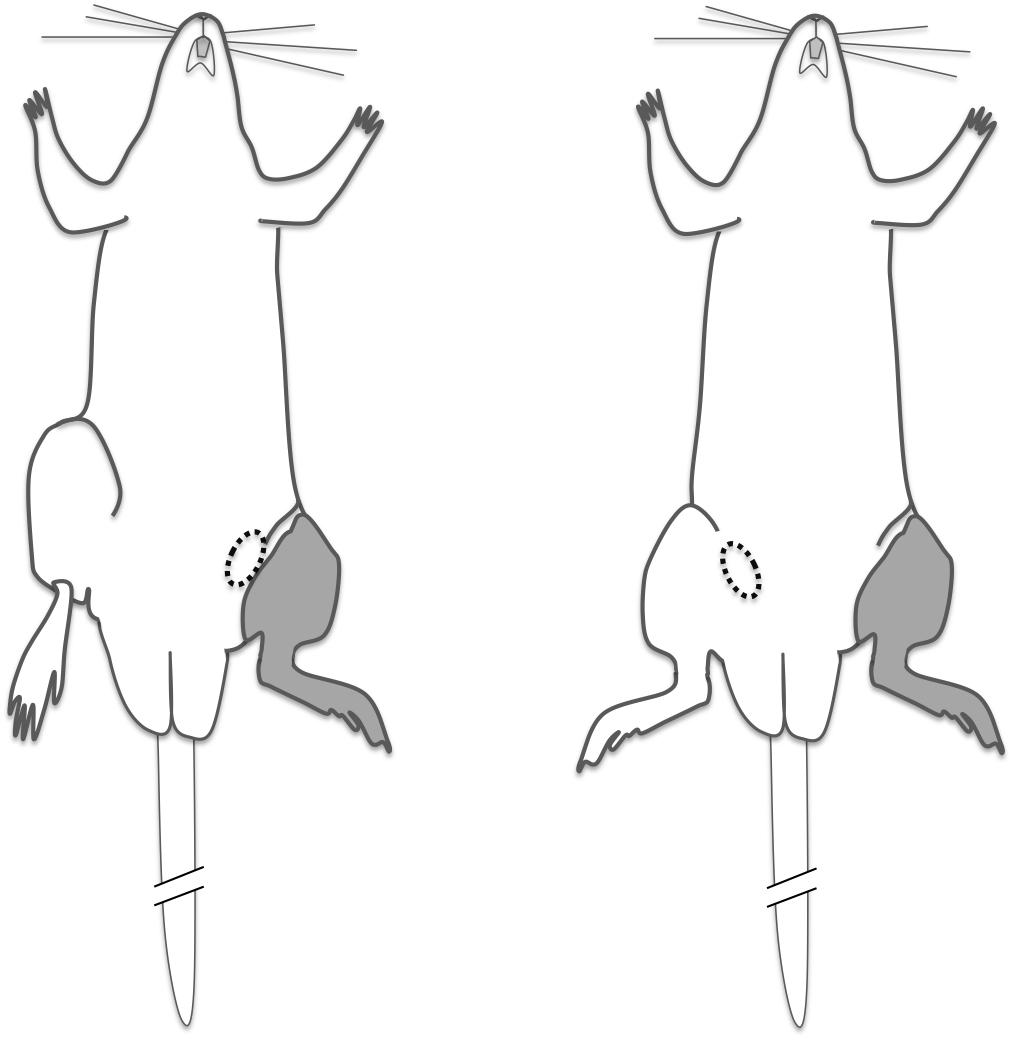
**

**B**

**
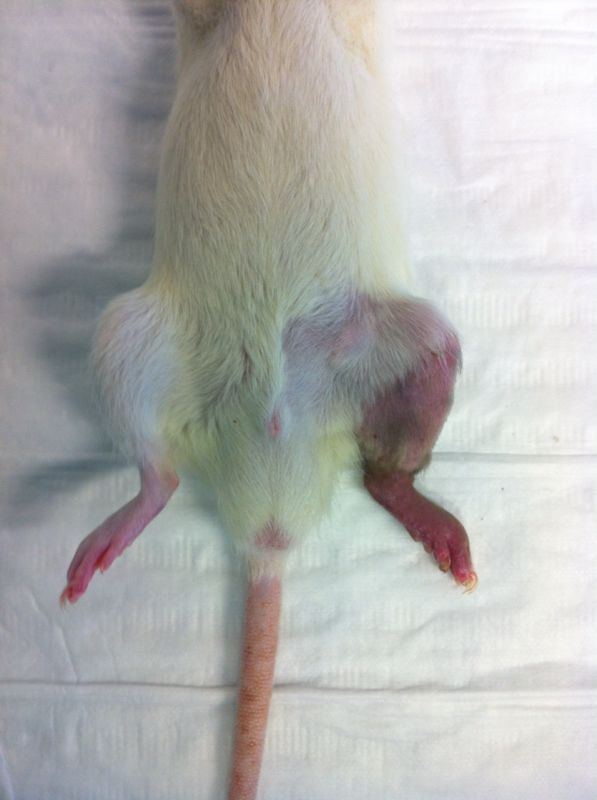
Supplementary Figure 1.** **Injection site of the Rapa-ISFI.** **A)** Schematic of the injection site of the Rapa-ISFI at post-operative day (POD7) in rat of Group 2 (injection in the groin close to the transplanted limb) and rats of Group 3 (injection in the groin of contralateral native leg). Circle represents the injection site for each group. **B**) Picture of the RAPA-ISFI after injection. A small swollen area (yellow circle) is visible under the skin after injection.

**
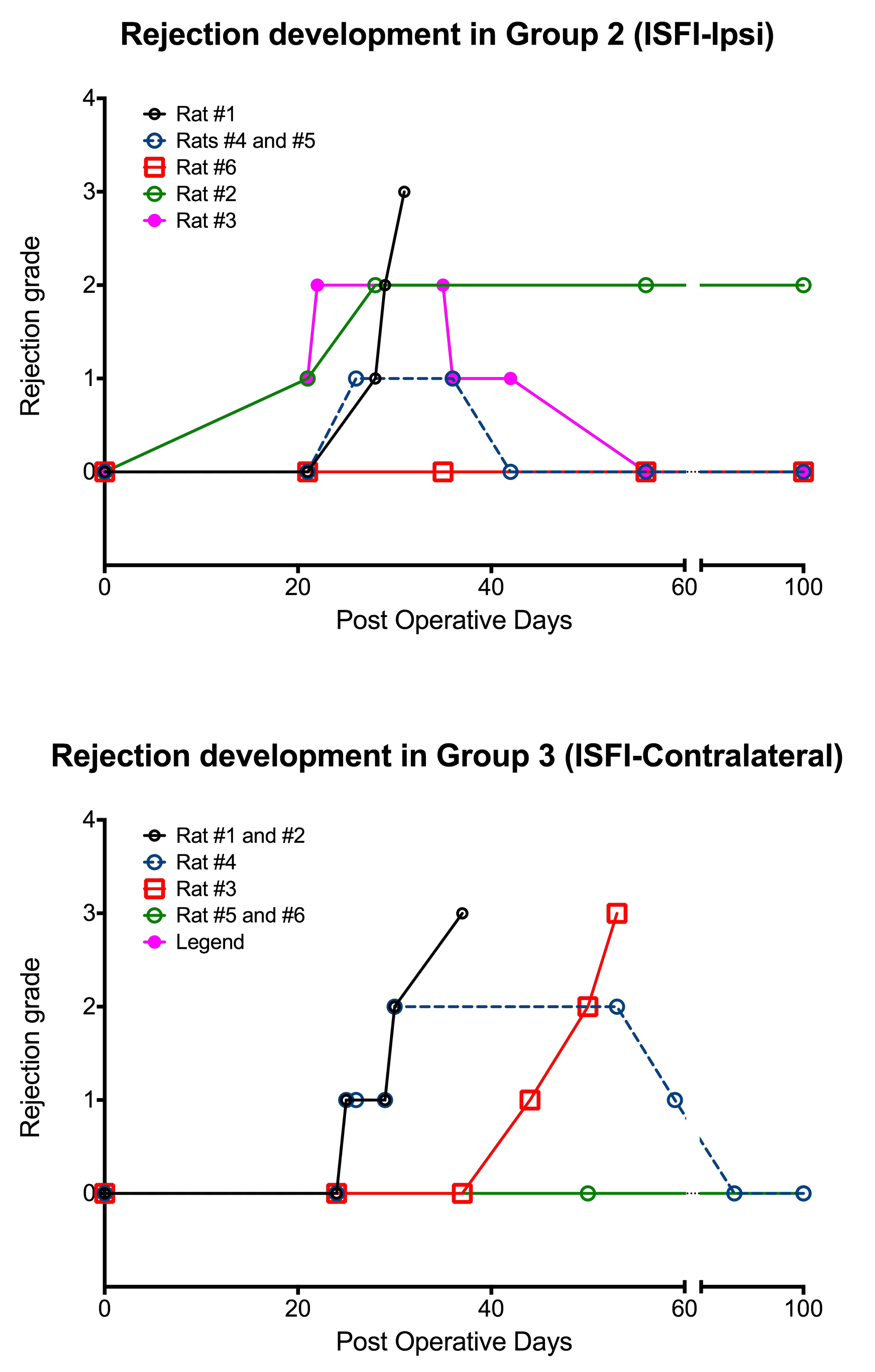
**

**Supplementary Figure 2. Evaluation of macroscopic rejection in Rapa-ISFI treated rats.** Development of rejection episodes in rats from Group 2 and 3. Graft rejection was graded daily as 0=no rejection, 1=erythema and edema, 2=epidermolysis and exudation, and 3=desquamation, necrosis, and mummification. Each line represents a single rat.


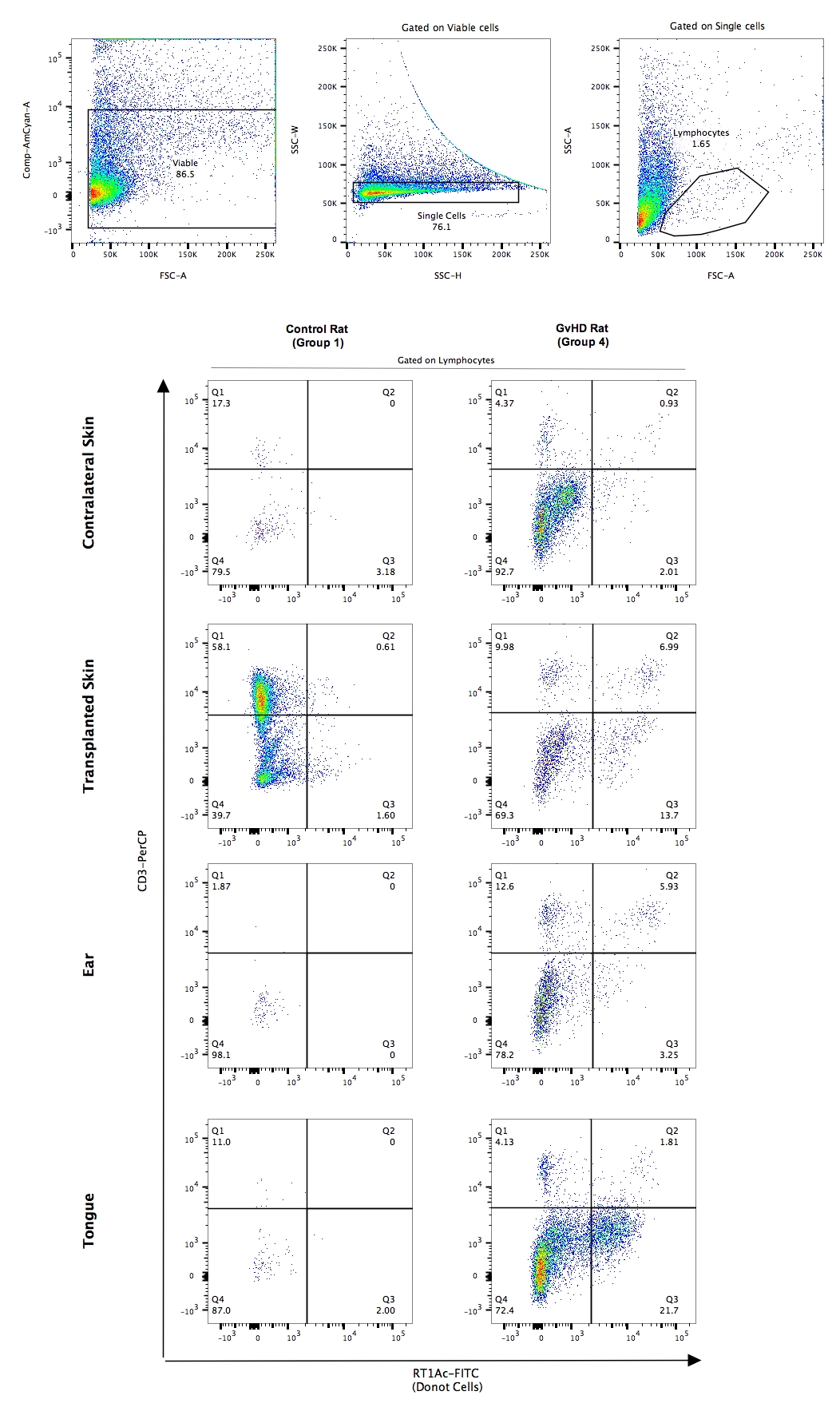
**Supplementary Figure 3. Donor lymphocyte infiltration in GvHD lesions.** Flow-cytometry analysis of the lymphocyte infiltration in GvHD lesions in recipient ear and tongue. Tissues were retrieved at sacrifice, digested and stained for flow-cytometry. In all the samples dead cells were excluded by Fixable Viability Die (in AmCyan Channel), then single cells were selected and lymphocyte population was chosen based on physical parameters (first row). Among the lymphocytes, T cells were identified as CD3+ cells and donor cells as RT1Ac+ cells. Untreated rats rejecting their graft from Group 1, presented a great number of recipient T cells (CD3+RT1AC- cells) in the transplanted skin and only a few in the contralateral skin, confirming the recipient T cell infiltration in the graft at rejection. In these rats the number of donor cells was relatively low in ear and tongue. Rats of Group 4 with macroscopic signs of GvHD showed clear infiltration of donor cells (RT1Ac+) and donor T cells (CD3+RT1Ac+) in the affected tissue (*i.e.,* ear and tongue) accompanied also by the infiltration of T cells of recipient origin due to strong inflammation. Representative pictures of 2 control rats and 3 rats with GvHD.


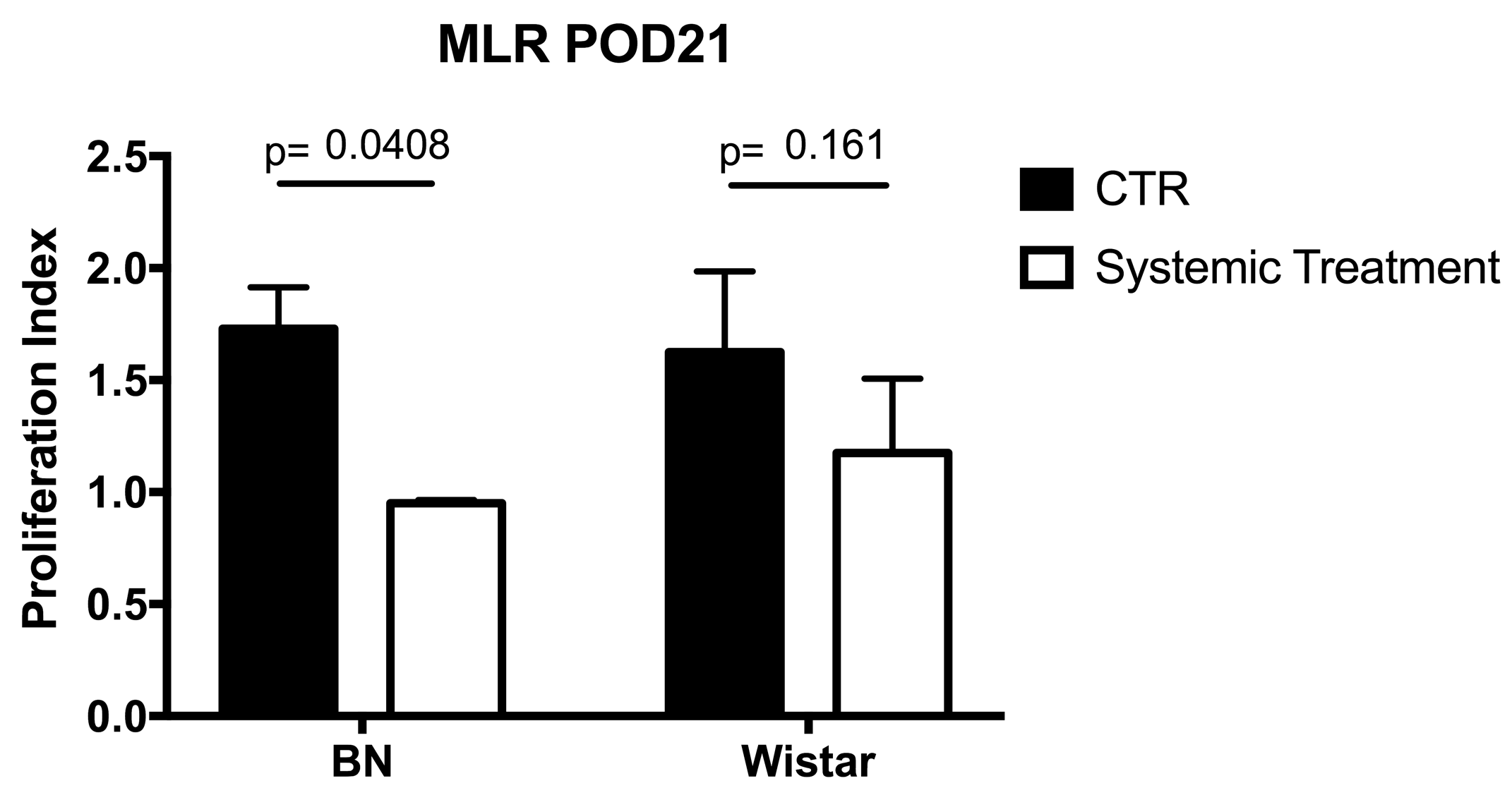


**Supplementary Figure 4. Rats with GvHD showed significantly reduced response to BN cells as compared to untreated rats.** Peripheral blood mononuclear cells were isolated at POD21 and stimulated either with donor cells (BN) or third party cells (Wistar). Proliferation was measured by evaluating CFSE dilution with Flow-Jo. Control rats from Group 1 (n=2) and rats from Group 4 treated with systemic rapamycin showing signs of GvHD (n=2) were compared. Data presented as mean and SD. P values were determined using the Holm-Sidak method.

**
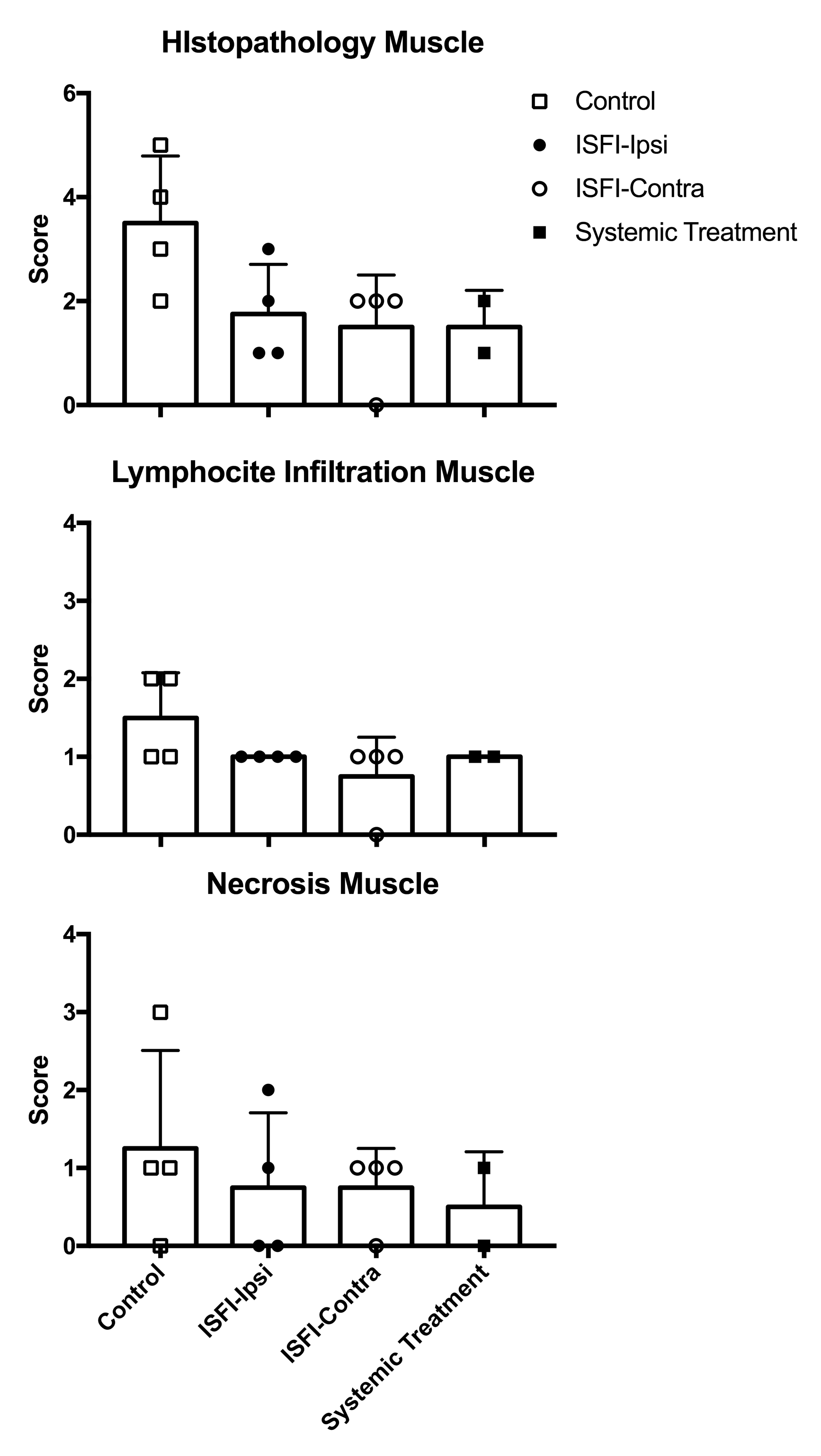
**

**Supplementary Figure 5. Muscle Histopathology.** Muscle samples from the grafts, retrieved at the end of the experiments, were fixed, stained with hematoxylin and eosin (H&E) and blindly scored. A score from 0 to 3 was given for necrosis/atrophy and lymphocyte infiltration (*i.e.*, 0=absent, 1=minimal, 2=moderate or 3=extensive), the sum of these two categories gave the final muscle histopathological score. Data are presented as mean and SD and analyzed by one-way ANOVA with Tukey’s multi-comparisons test.


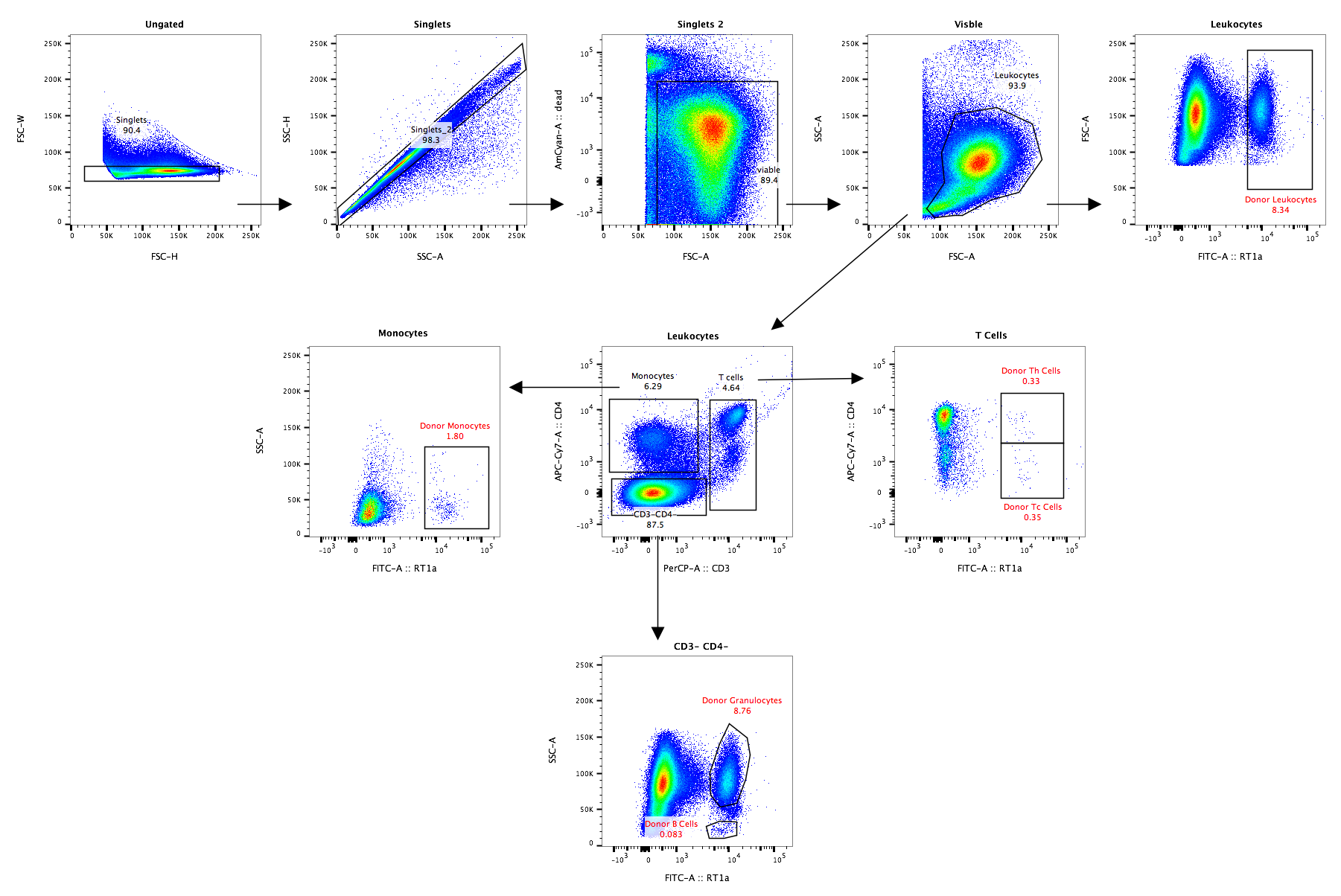


**Supplementary Figure 6 (previous page). Gating strategy for the quantification of multilineage chimerism levels in the peripheral blood of recipient rats.** Representative gating strategy for the quantification of multilineage chimerism levels in the peripheral blood of recipient rats at different time points after hind limb transplantation. After exclusion of doublets, dead cells and debris, donor cells (red names) were identified and quantified as frequency of circulating leukocytes. Positivity for the RT1Ac markers was set using naïve Lewis blood stained with the same panel as negative control. Donor leukocytes were identified as RT1Ac+ cells in the leukocytes gate; donor monocytes as CD3-CD4+RT1Ac+ leukocytes; donor T helper (Th) cells as CD3+CD4+ RT1Ac+ leukocytes; donor T cytotoxic (Tc) cells as CD3+CD4- RT1Ac+ leukocytes. Donor B cells and granulocytes were identified in the CD3-CD4- fraction based on their granularity (*i.e.*, side scatter) as CD3-CD4-SSC^low^RT1Ac+and CD3-CD4-SSC^high^RT1Ac+ leukocytes, respectively.

**
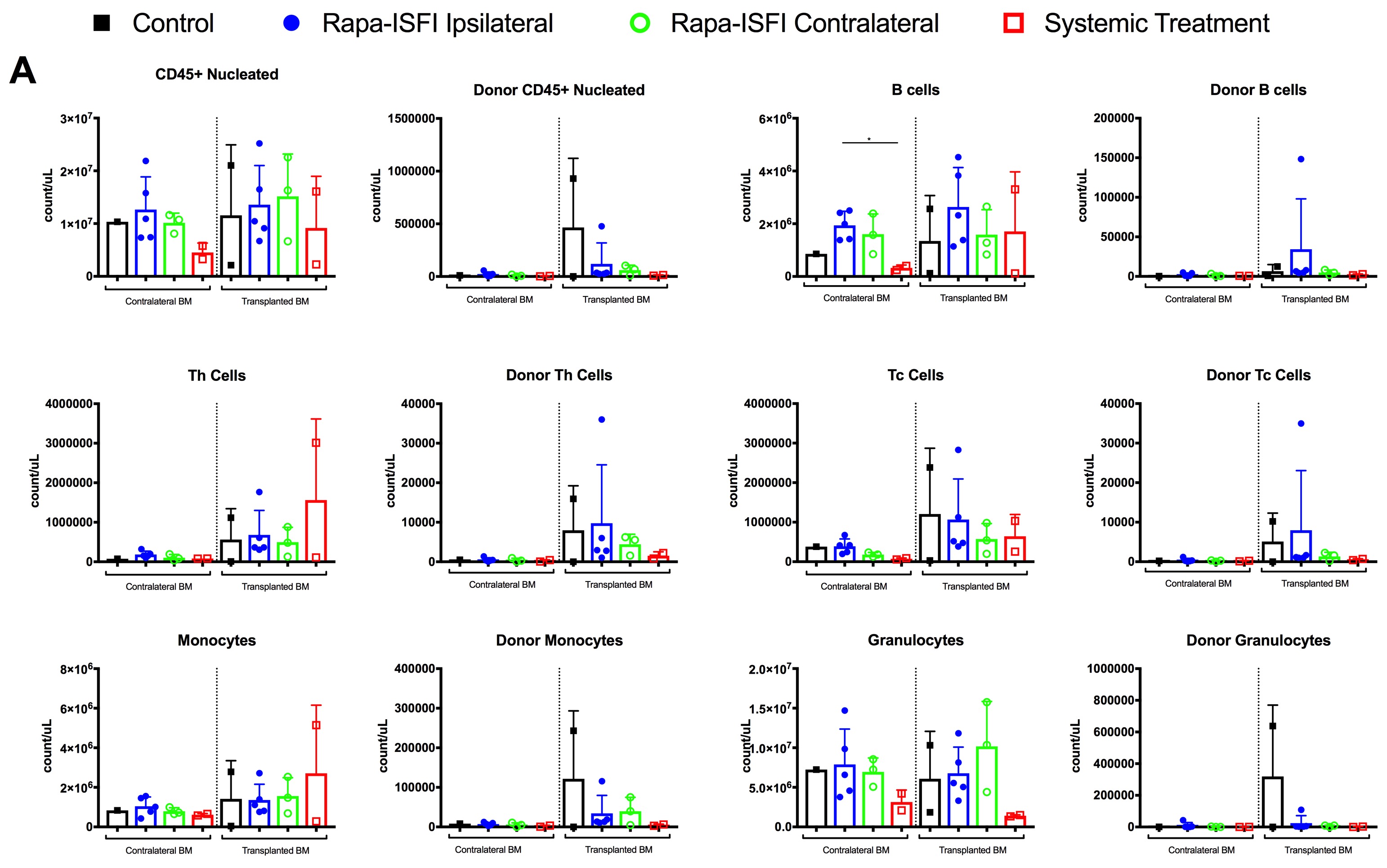
**

**
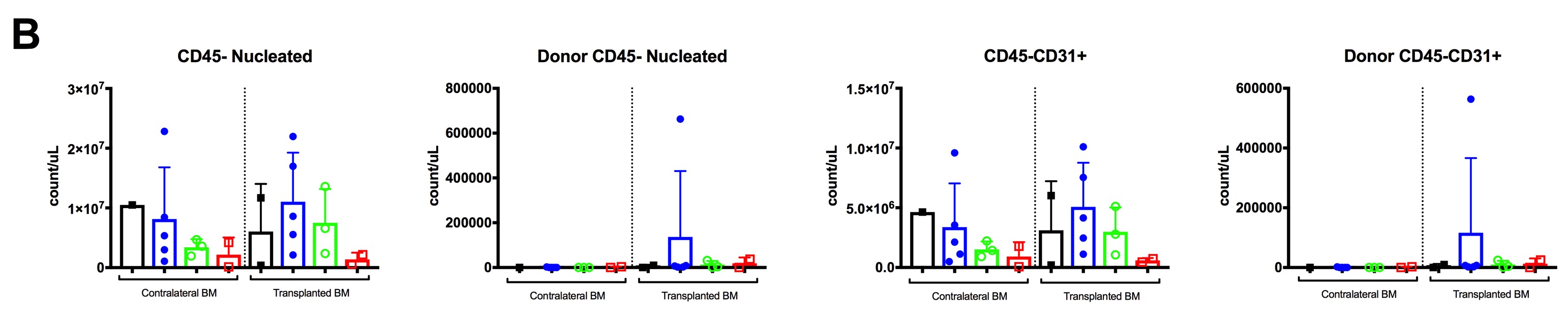
 Supplementary Figure 7: Bone Marrow characterization.** Bone marrow from the contralateral (i.e. recipient) and transplanted (i.e. donor bone) tibia was isolated and characterized by flow cytometry. **A) (previous page)** Absolute number of CD45+ expressing cells and their subpopulation at the end point. Viable, nucleated cells were identified using Hoechst 33342 staining and then or CD45+ were gated. Within this gate, B cells were identified as CD3^-^CD4^-^ FSc^Low^SSc^Low^CD45R^+^ cells, T helper (Th) cells as CD3^+^CD4^+^ cells, T cytotoxic (Tc) cells as CD3^+^CD4^-^cells, monocytes as CD3^-^CD4^+^ cells and granulocytes as CD3^-^CD4^-^FSc^High^SSc^High^ cells. The respective donor-derived cells were identified by the expression of the RT1Ac^+^ marker. **B**) Absolute number of CD45 negative cells and their subpopulation at the end point. Viable, nucleated cells were identified using Hoechst 33342 staining and then or CD45- were gated. Within this gate, CD31 expression was evaluated and the respective donor-derived cells were identified by the expression of the RT1Ac^+^ marker. Data expressed as cell/µL and presented as mean and SD, *P<0.05 by one-way ANOVA with Tukey’s multi-comparisons test.

**Supplementary Figure 8: Quantification of bone marrow stem cells.** Bone marrow from the contralateral of transplanted (i.e. donor bone) tibia was isolated and characterized by flow cytometry. Absolute number of stem cells identified as nucleated (Hoechst 33342 positive) cells negative for lineage (lin) markers (i.e. CD3-CD4-CD45R-) CD45+ CD34+ and/or CD133 or nucleated CD45- CD34+ and/or CD133+ cells. The respective donor-derived cells were identified by the expression of the RT1Ac^+^ marker. Data expressed as cell/µL and presented as mean and SD. **
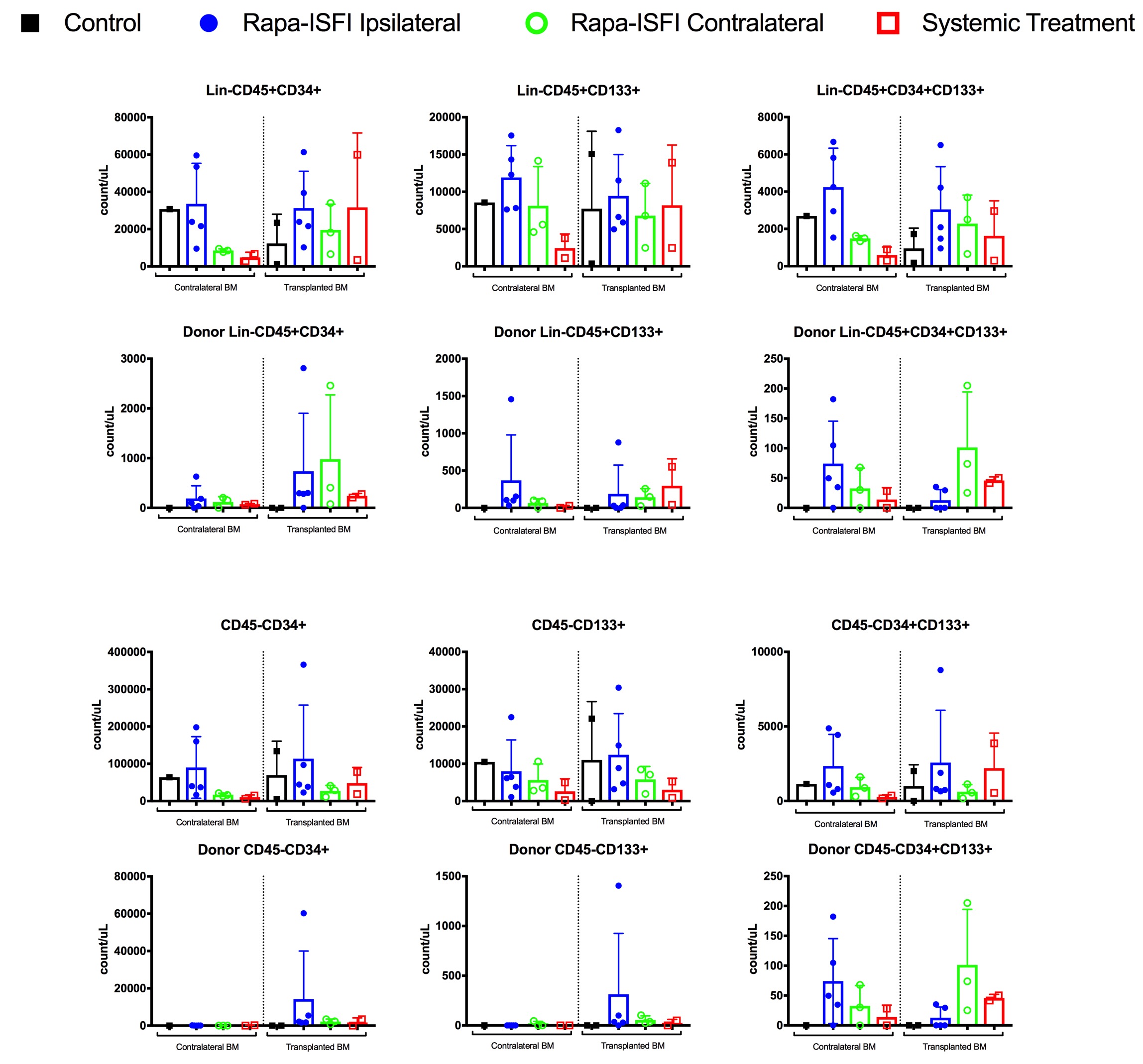
**

**
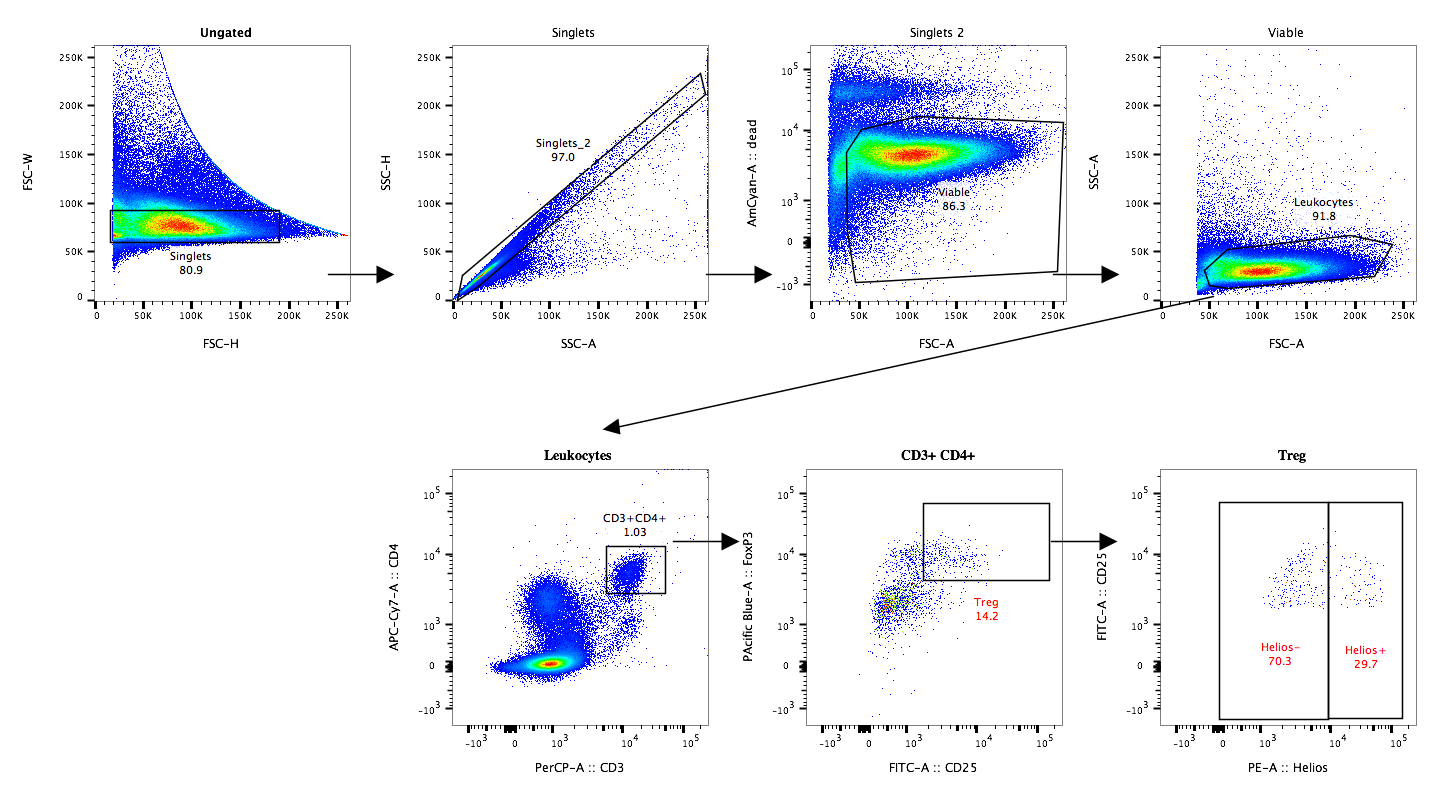
**

**Supplementary Figure 9 (previous page). Gating strategy for the quantification of T_reg_, Helios^Pos^ and Helios^Neg^ T_reg_ in the peripheral blood.** T_reg_ were identified as CD3+CD4+CD25+FoxP3+ cells after exclusion of doublets, dead cells ad debries. Helios^Pos^ and Helios^Neg^ cells were identified among the T_reg_ cells based on the expression of the transcription factor Helios. CD25 and FoxP3 positivity was set based on fluorescence minus one (FMO) controls. The frequency of all the T_reg,_ Helios^Pos^ T_reg_ and Helios^Neg^ T_reg_ was expressed as % of CD3+CD4+.

**
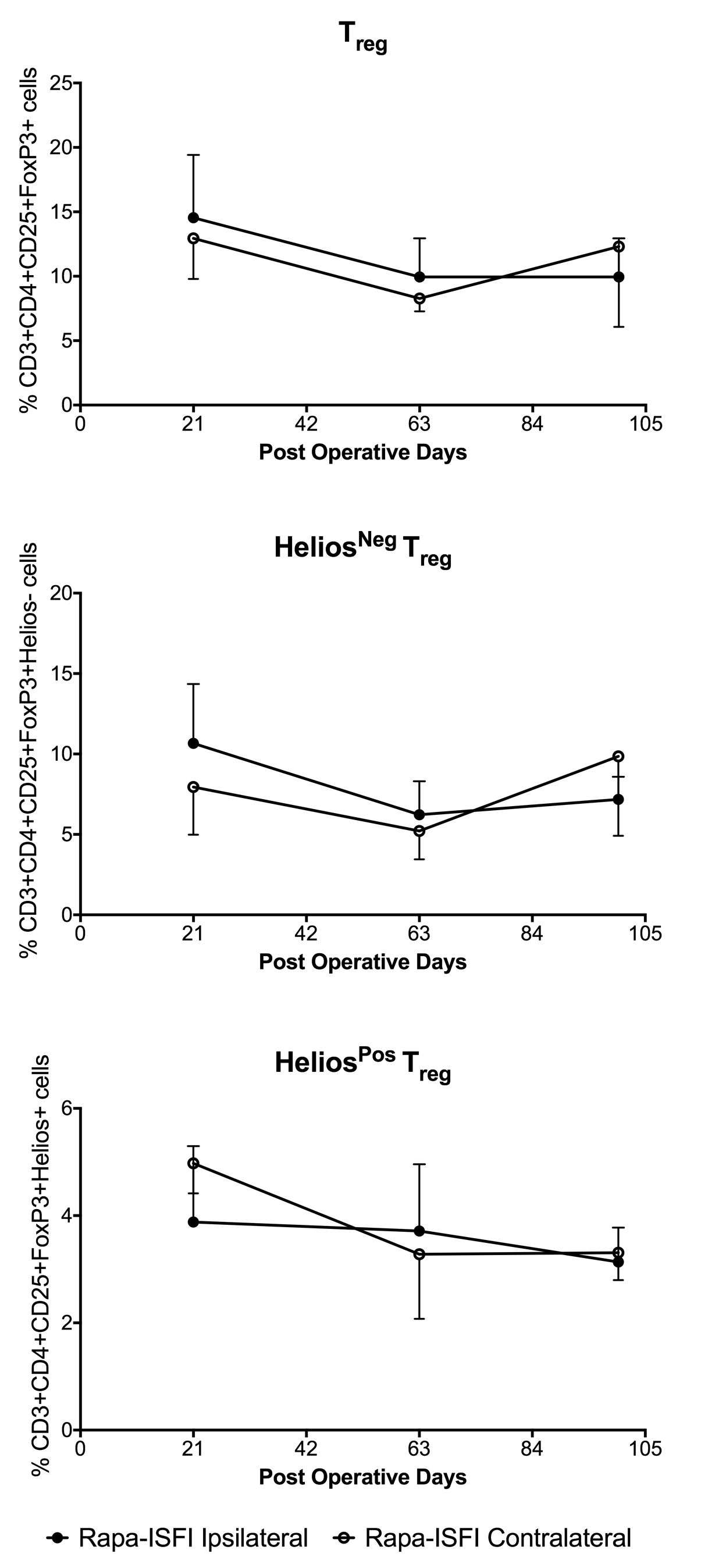
Supplementary Figure 10. The frequency of T_reg_, Helios^Pos^ and Helios^Neg^ T_reg_ in the peripheral blood of the recipient rats treated with Rapa-ISFI remains stable during the experiment.** T_reg_, Helios^Neg^ T_reg_ and Helios^Pos^ T_reg_ were quantified at POD21, 63 and 100 in the peripheral blood of rats of Groups 2 (Rapa-ISFI Ipsilateral, black circles) and 3 (Rapa-ISFI-Cotralateral, open circle) as described. Data presented as mean and SD.


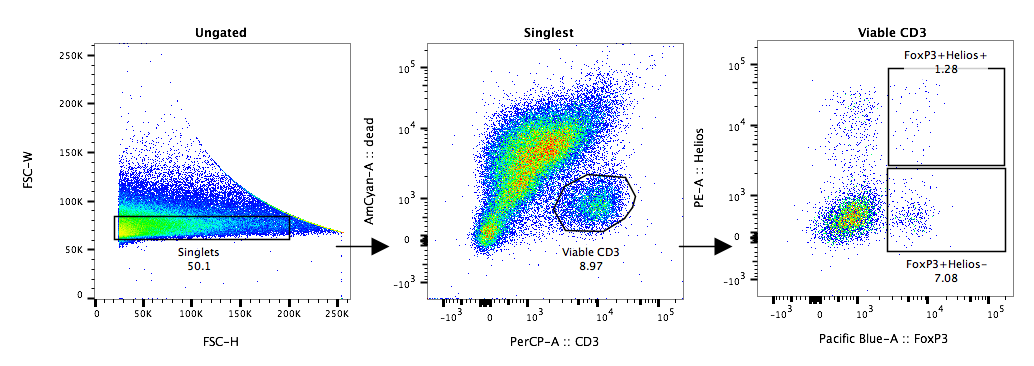


**Supplementary Figure 11. Gating strategy for quantification of T_reg_, Helios^Pos^ and Helios^Neg^ T_reg_ in the skin.** Representative gating strategy for the enumeration of T_reg_ in the skin collected from VCA transplant. T_reg_ were identified as FoxP3+ cells after exclusion of doublets and selection of viable CD3+ cells. Helios^Pos^ and Helios^Neg^ cells were identified among the CD3+FoxP3+ T_reg_ based on the expression of the transcription factor Helios. The frequency of all the T_reg_ population was expressed as % Viable CD3+ cells.

## Supplementary References

1. Karfeld-Sulzer LS, Ghayor C, Siegenthaler B, de Wild M, Leroux J-C, Weber FE. N-methyl pyrrolidone/bone morphogenetic protein-2 double delivery with in situ forming implants. *J Control Release* (2015) **203**:181–188. doi:10.1016/j.jconrel.2015.02.019

2. Sacks JM, Kuo Y-R, Horibe EK, Hautz T, Mohan K, Valerio IL, Lee WPA. An optimized dual-surgeon simultaneous orthotopic hind-limb allotransplantation model in rats. *Journal of Reconstructive Microsurgery* (2012) **28**:69–75. doi:10.1055/s-0031-1285822

3. Gajanayake T, Olariu R, Leclère FM, Dhayani A, Yang Z, Bongoni AK, Banz Y, Constantinescu MA, Karp JM, Vemula PK, et al. A single localized dose of enzyme-responsive hydrogel improves long-term survival of a vascularized composite allograft. *Science Translational Medicine* (2014) **6**:249ra110. doi:10.1126/scitranslmed.3008778

4. Sucher R, Oberhuber R, Margreiter C, Rumberg G, Jindal R, Lee WPA, Margreiter R, Pratschke J, Schneeberger S, Brandacher G. Orthotopic hind-limb transplantation in rats. *J Vis Exp* (2010) doi:10.3791/2022

5. Olariu R, Denoyelle J, Leclère FM, Dzhonova DV, Gajanayake T, Banz Y, Hayoz M, Constantinescu M, Rieben R, Vögelin E, et al. Intra-graft injection of tacrolimus promotes survival of vascularized composite allotransplantation. *Journal of Surgical Research* (2017) **218**:49–57. doi:http://dx.doi.org/10.1016/j.jss.2017.05.046

6. Cendales LC, Kanitakis J, Schneeberger S, Burns C, Ruiz P, Landin L, Remmelink M, Hewitt CW, Landgren T, Lyons B, et al. The Banff 2007 working classification of skin-containing composite tissue allograft pathology. in (Blackwell Publishing Ltd), 1396–1400. doi:10.1111/j.1600-6143.2008.02243.x
